# Metabolic Profiling Reveals Biochemical Pathways Responsible for Eelgrass Response to Elevated CO_2_ and Temperature

**DOI:** 10.1038/s41598-020-61684-x

**Published:** 2020-03-13

**Authors:** Carmen C. Zayas-Santiago, Albert Rivas-Ubach, Li-Jung Kuo, Nicholas D. Ward, Richard C. Zimmerman

**Affiliations:** 1 Department of Ocean, Earth & Atmospheric Sciences, Old Dominion University, Norfolk, VA 23429 USA; 2 Environmental Molecular Sciences Division, Pacific Northwest National Laboratory, 3335 Innovation Boulevard, Richland, WA 99352 USA; 3 Marine Sciences Laboratory, Pacific Northwest National Laboratory, 1529 W. Sequim Bay Rd., Sequim, WA 98382 USA; 4 School of Oceanography, University of Washington, Seattle, WA 98105 USA

**Keywords:** Plant molecular biology, Climate-change ecology, Environmental impact

## Abstract

As CO_2_ levels in Earth’s atmosphere and oceans steadily rise, varying organismal responses may produce ecological losers and winners. Increased ocean CO_2_ can enhance seagrass productivity and thermal tolerance, providing some compensation for climate warming. However, the metabolic shifts driving the positive response to elevated CO_2_ by these important ecosystem engineers remain unknown. We analyzed whole-plant performance and metabolic profiles of two geographically distinct eelgrass (*Zostera marina* L.) populations in response to CO_2_ enrichment. In addition to enhancing overall plant size, growth and survival, CO_2_ enrichment increased the abundance of Calvin Cycle and nitrogen assimilation metabolites while suppressing the abundance of stress-related metabolites. Overall metabolome differences between populations suggest that some eelgrass phenotypes may be better suited than others to cope with an increasingly hot and sour sea. Our results suggest that seagrass populations will respond variably, but overall positively, to increasing CO_2_ concentrations, generating negative feedbacks to climate change.

## Introduction

In addition to being a climate warming greenhouse gas, nearly half of the anthropogenically released carbon dioxide (CO_2_) is absorbed by the ocean, eliciting both negative and positive organismal responses to the process known as Ocean Acidification^1,2^. Benthic calcifiers, including corals and mollusks, are expected to respond negatively as calcification becomes energetically more expensive in an acidified ocean^3^. However, CO_2_ is also a potentially limiting substrate for photosynthesis in both terrestrial and aquatic ecosystems. Terrestrial plants grown under CO_2_ enrichment often show increased rates of carbon uptake, sucrose formation, nitrogen and water use efficiencies, dark respiration and growth^4^. Although many marine autotrophs exhibit very little response to CO_2_ fertilization^5^, some nitrogen-fixing cyanobacteria^6^, coccolithophores^7^, chlorophytes such as *Caulerpa* spp. and *Ulva* spp.^8,9^, and especially seagrasses^10–12^ exhibit increased rates of photosynthesis, growth and biomass production under CO_2_ enrichment.

Seagrasses are well recognized as important ecosystem engineers^13^ and Blue Carbon reservoirs^14^, but their populations are increasingly threatened by anthropogenic degradation of water quality and climate warming^15^. Experimental^16^ and theoretical^17^ evidence indicates that enhanced photosynthesis stimulated by rising CO_2_ availability can offset the effects of thermal stress for eelgrass (*Zostera marina* L.); a problem that appears to be increasing with climate warming^18–20^. Eelgrass, the most widely distributed seagrass species in temperate marine environments of the Northern Hemisphere, persists in geographically isolated populations spanning different thermal environments from the subarctic Bering Sea to the seasonally warm waters of the mid-Atlantic Bight and Mediterranean^21^. This distribution lends eelgrass to be the most extensively studied seagrass species in terms of ecology, physiology and genetics, making it a unique model organism for exploring the impacts of climate change on seagrass ecosystems. In addition, seagrass meadows are among the most productive aquatic habitats in terms of carbon burial^22^, suggesting that enhanced seagrass productivity under increasing CO_2_ conditions may exert a negative feedback on climate change.

Eelgrass exhibits a wide range of morphological variation throughout its geographic distribution that has been related to local environmental conditions, including substrate type, water depth, temperature, light, nutrient availability and water flow^23^ and inherent genetic variation^24^. These circumstances suggest populations may have had numerous opportunities for genetic adaptation to different environments, making eelgrass an ideal species for exploring the impacts of climate change across geographic regions. However, the degree of morphological and physiological plasticity within geographically isolated populations remains unknown. Heat stress in European populations of *Z. marina* and *Z. noltii* appears to be mediated primarily by its effect on sucrose metabolism^25^. Consequently, photosynthetic stimulation resulting from CO_2_ enrichment increases sucrose formation and should reduce the effects of thermal stress. Prolonged exposure to elevated CO_2_ quantitatively enhances leaf photosynthesis, shoot survival, growth and flowering of eelgrass populations from climates characterized by a narrow thermal range (predominantly cool)^16,26^ and a wide thermal range that include stressfully warm summers^11^, thereby demonstrating that eelgrass productivity and thermal tolerance in the modern-day ocean are strongly mediated by CO_2_ availability.

Here we evaluated the physiological and metabolome responses of two distinct eelgrass populations from Puget Sound, Washington and Chesapeake Bay, Virginia, USA that represent contrasting thermal environments. These populations were subjected to an experimental gradient of five CO_2_ conditions in an outdoor facility under naturally varying temperature and insolation for one year. Accordingly, we compared the plants physiological processes in response to the environment and characterized the metabolome of the samples at the end of the 1-year exposure period. The metabolome of an organism is considered its chemical phenotype^27^, as it is the first component responding to external stressors^28^. The metabolome consists of thousands of low molecular weight metabolites (typically < 800 Da) such as amino acids, organic acids, sugars and phenolic compounds derived from primary and secondary cellular metabolism. This study explored the hypothesis that increased CO_2_ availability would stimulate carbon fixation pathways and reduce the biosynthesis of stress-related compounds. Differential responses among populations may help identify heritable traits that facilitate adaptation of eelgrass to a changing climate and improve our predictive capacity for restoring and conserving these important ecosystem engineers.

## Morphology and whole plant performance

**Figure 1:**
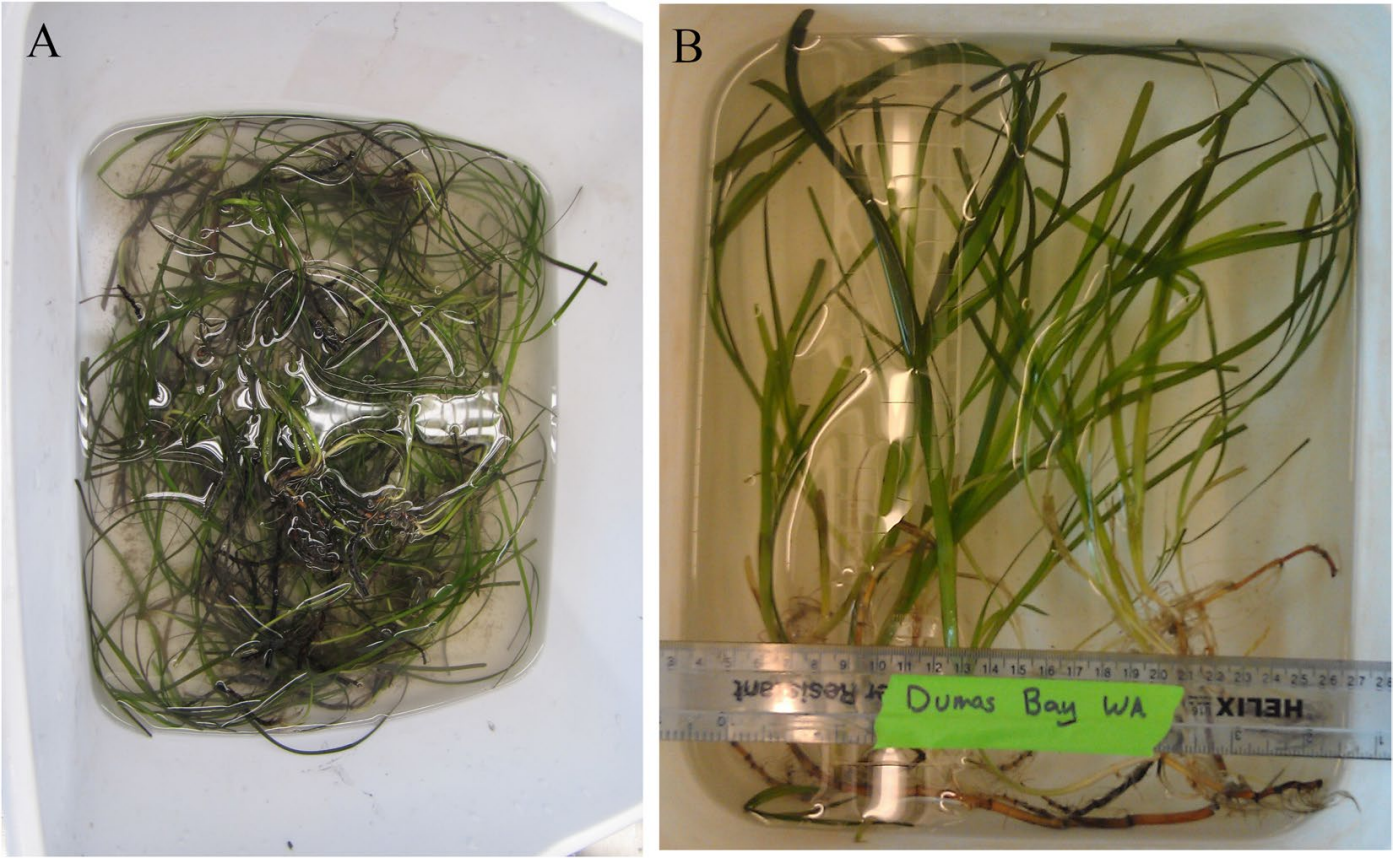
Photographs of eelgrass from (**A**) South Bay, VA (left) and (**B**) Dumas Bay, WA (right) showing morphological differences at the time of original collection.

**Figure 2:**
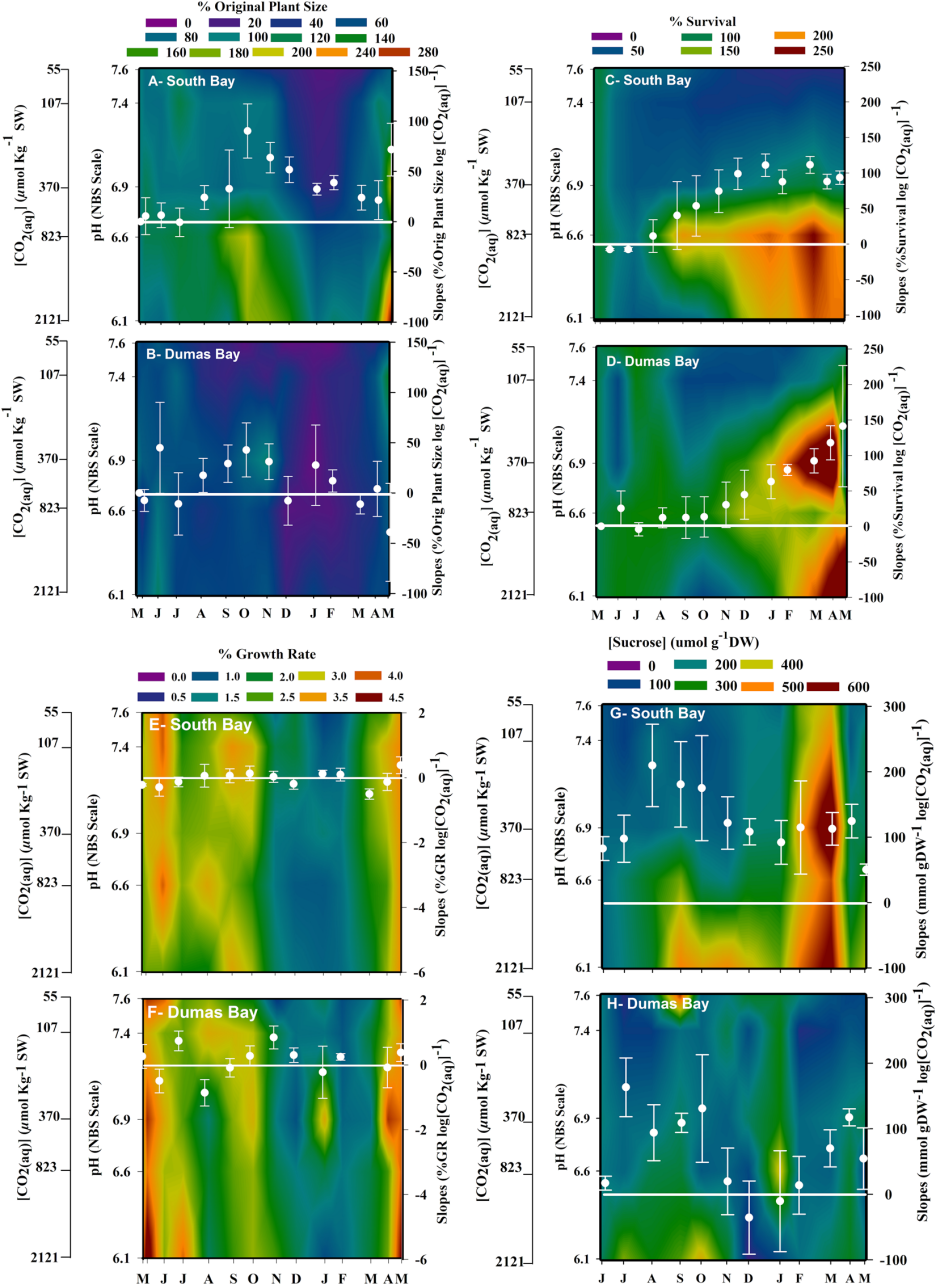
Heat maps showing the responses of South Bay VA and Dumas Bay WA eelgrass populations to [CO_2_] and time. (**A**,**B**) Percent plant size. (**C**,**D**) Percent shoot survival. (**E**,**F**) Relative growth rate. (**G**,**H**) Leaf sucrose concentration. Tick marks on the left vertical axis of each plot indicate the mean [CO_2_] for each treatment. White symbols on each plot represent the monthly slope of the response variable vs. log [CO_2_] derived from linear regression analysis. Error bars represent ± 1 SE of the regression slope. South Bay data adapted from^11^.

At the start of this experiment, eelgrass (*Zostera marina* L.) shoots from Puget Sound (Dumas Bay, WA, USA) were significantly longer and wider, reaching up to 1 m in length and 0.3–0.5 cm in width, than eelgrass from the Chesapeake region (South Bay, VA, USA) that grow to about 30 cm in length and 0.1–0.5 cm in width (Fig. 1). CO_2_ enrichment yielded strong positive effects on individual shoot size, vegetative shoot numbers (shown as % survival) and sucrose content of both populations during the 12-month experiment (Fig. 2). However, plants from South Bay, VA (SBV) showed larger increases in size compared to those from Dumas Bay, WA (DBW), especially during summer and early fall 2013 (Fig. 2A,B and Table S1). The stimulating effects of CO_2_ enrichment on plant size of both populations decreased during the winter period of low light availability and low temperature (Fig. 2A,B white symbols and lines).

High [CO_2_] stimulated vegetative shoot survival in both eelgrass populations into the fall of 2013 and especially through the winter of 2013–2014 (Fig. 2C,D). However, shoot numbers decreased under ambient [CO_2_] for both eelgrass populations during the summer period of warm (>25 °C) water temperature. Shoot losses continued under ambient [CO_2_] as water temperature dropped throughout the fall 2013 and into the winter of 2014. The effect of CO_2_ on shoot survival, as indicated by the slope of percent survival vs. log [CO_2_], was highest from winter (January) to spring (May) 2014 for SBV plants and from early (March) to late (May) spring 2014 for DBW plants (Fig. 2C,D white symbols and lines, Table S2). At the end of the experiment (May 2014), shoot numbers in the highest CO_2_ treatment doubled through vegetative proliferation but less than half originally transplanted shoots survived under ambient [CO_2_].

Although plant size and therefore absolute growth rate increased with [CO_2_], there was no CO_2_ effect on relative growth rate (Fig. 2E,F). Despite CO_2_ enrichment, both SBV and DBW populations increased growth rates during summer and early fall of 2013, even when water temperature exceeded the 25 °C threshold for eelgrass heat stress^18,29,30^. Growth rates of both populations declined during winter period of low light availability and temperature in all CO_2_ treatments (Fig. 2E,F) but recovered as temperature and light availability increased during spring 2014. The monthly trends of relative shoot growth (but not absolute growth) were significantly different between populations at different time points. For example, in late summer (August) and late winter (January) SBV showed higher slopes but DBW slopes were higher in fall (November) and winter (December), (Fig. 2E,F, white symbols and lines, Table S3).

Leaf sugar concentrations peaked in all CO_2_ treatments for both eelgrass populations in late winter (Fig. 2G,H), when temperature (Fig. S1) and growth were low. Sucrose concentrations of SBV leaves were higher than those from DBW during the whole experiment in high [CO_2_]. The monthly slopes of leaf sucrose concentration vs. log [CO_2_] were significantly different between eelgrass populations, SBV presented higher slopes (Fig. 2G,H, white symbols and lines, Table S4) in late summer (August), fall (November) and early winter (December).

## Metabolomic Response of Eelgrass

### Comparison between populations at high and low CO_2_

**Figure 3:**
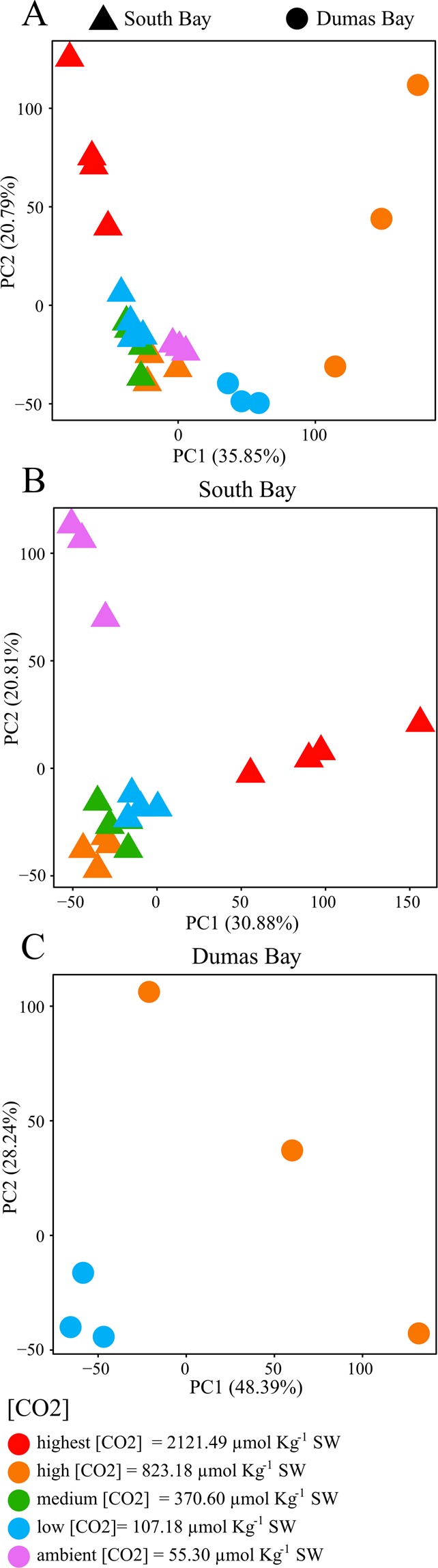
Principal Component Analyses of the metabolome fingerprints of eelgrass leaves from May 2014 growing at different CO_2_ concentrations from South Bay, VA (triangles) and Dumas Bay (circles), (**A**) together, (**B**) South Bay separately, and (**C**) Dumas Bay separately. CO_2_ treatment is indicated by color.

Permutational multivariate analysis of variance (PERMANOVA) of the entire metabolomic fingerprints, including both populations and CO_2_, showed overall significant differences between the SBV and DBW populations after 1-year growth in the experimental aquaria (Table S5). However, the interaction between CO_2_ treatment and population was not statistically significant (p = 0.077) (Table S5), suggesting that both populations showed similar responses to elevated CO_2_. Individual one-way ANOVAs for each metabolite detected significant differences (*p* < 0.05) between SBV and DBW plants in the abundance of some primary metabolites (Glycolysis – Krebs – Calvin) across CO_2_ treatments (Tables S6 and S7). Principal component analysis (PCA) including the whole eelgrass metabolomic fingerprints clearly clustered the two populations along the first Principal Component Axis 1(PC1) (Fig. 3A), with CO_2_ treatments separated along the PC2 showing differences between plants growing at different [CO_2_].

**Figure 4:**
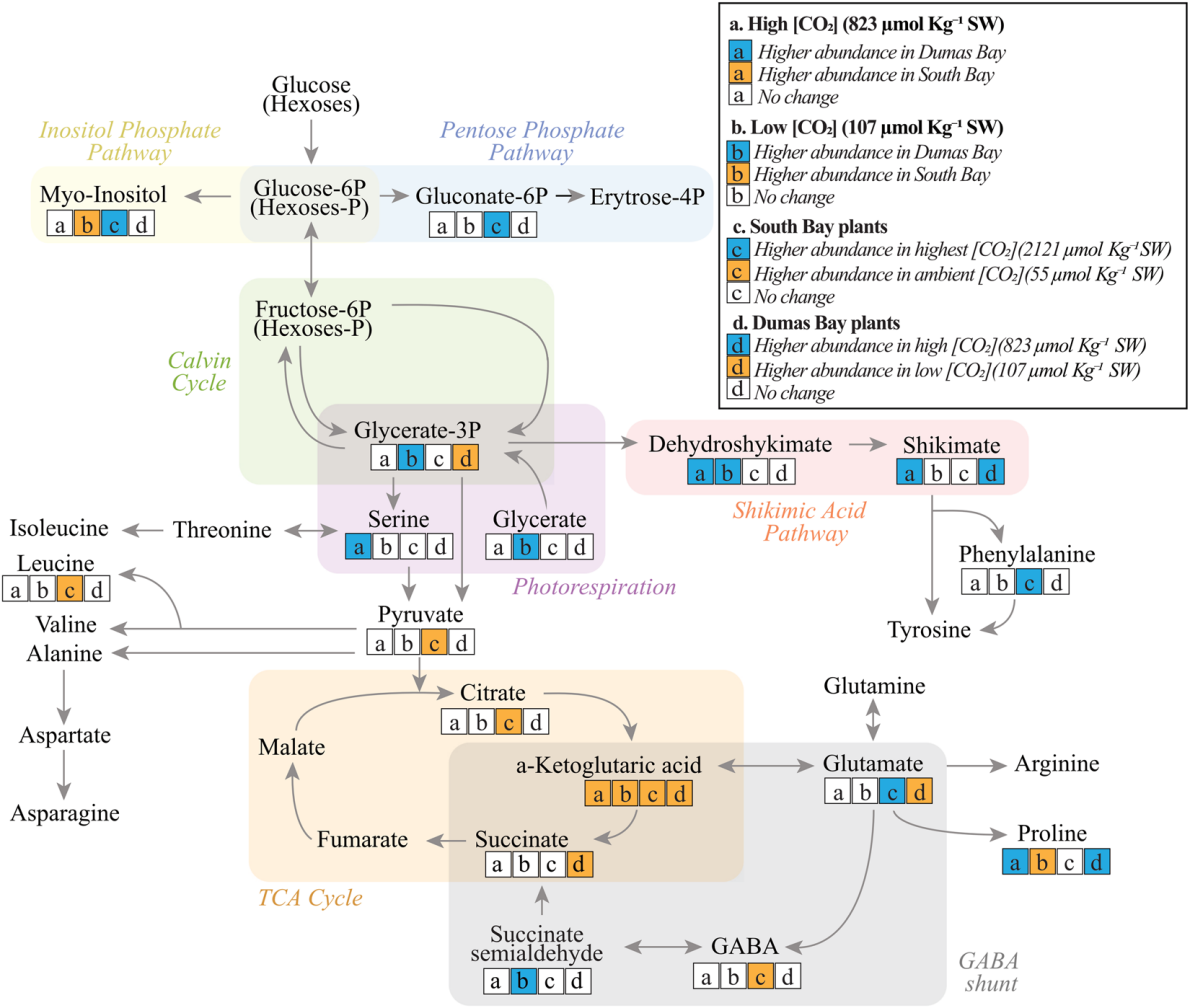
Representation of the main metabolic pathways of *Z. marina* from South Bay VA and Dumas Bay WA in response to high and low CO_2_ concentrations. Only identified metabolites are represented in the schema. Significant changes in any of the metabolite comparisons are represented in bold typeface. Colored boxes below metabolite names represent the result of each of the comparisons after one-way ANOVA. Each letter within each box represent a different comparison: (**a**) South Bay vs. Dumas Bay plants growing at high CO_2_ (823 μmol CO_2_ kg^−1^SW). (**b**) South Bay vs. Dumas Bay plants growing at low CO_2_ (107 μmol CO_2_ kg^−1^SW). For a and b, blue and orange colors indicate higher relative abundance in Dumas Bay and South Bay plants, respectively. (**c**) Highest vs. ambient CO_2_ conditions (2121 vs 55 μmol CO_2_ kg^−1^SW) plants from South Bay (**d**) High vs. low CO_2_ conditions (823 vs 107 μmol CO_2_ kg^−1^ SW) plants from Dumas Bay. For c and d, blue and orange color indicate higher relative abundance of metabolites in plants growing at high CO_2_ (2121 μmol CO_2_ kg^−1^ SW in **c**, 823 μmol CO_2_ kg^−1^ SW in **d**) and ambient or low CO_2_ (55 μmol CO_2_ kg^−1^ SW in **c**, pH 107 μmol CO_2_ kg^−1^ SW in **d**), respectively.

Although CO_2_ enhanced growth, survival and thermal tolerance of both eelgrass populations, DBW contained higher abundances of photorespiratory and stress-related compounds in the shikimate pathway than SBV regardless of CO_2_ treatments (Fig. 4a,b, Tables S6, S7). Higher abundance of dehydroshikimate (Fig. 4a,b, Tables S6, S7) observed in DBW plants, relative to SBV, may indicate up-regulation of metabolic flux through the shikimate pathway^31^ leading to the synthesis of polyphenols. Stress conditions such as high light and pathogens^32^, and low CO_2_^33^ increase the biosynthesis of phenolic compounds in seagrasses.  Further, the shikimic intermediates are known to respond to oxidative stress and copper pollution in some macrophytes^34,35^. On the other hand, SBV plants had higher abundances of α-ketoglutaric acid (TCA Cycle) across CO_2_ treatments (Fig. 4a,b, Tables S6, S7). Studies have reported accumulation of α-ketoglutaric acid under oxidative stress in *Z. marina*^36^ and rice^37^ and have been suggested as a mitigation mechanisms to manage stressful events limiting the accumulation of pyruvate^36^.

At high [CO_2_], proline and serine were more abundant in DBW eelgrass than in SBV (Fig. 4a, Table S6). Proline is known to aid stress tolerance by acting as a metal chelator, by providing antioxidative defense and as a signaling molecule^38,39^ to control mitochondrial functions and, other developmental processes, and activate gene expression that may facilitate plant recovery from stress^40^. Serine has also been related to stress tolerance (e.g., low temperature and elevated salinity in *Arabidopsis thaliana*^41^ and references therein) and is synthesized (i) through the photorespiratory glycolate pathway, (ii) from Calvin Cycle intermediates (the “phosphorylated” pathway) and/or (iii) the glycerate pathway via cytosolic glycolysis^42^. However, high [CO_2_] is known to decrease photorespiration in eelgrass^43^, suggesting that the elevated abundance of serine observed here were likely being driven by non-photorespiratory pathways.

Metabolites involved in biotic/abiotic stress responses were elevated in both populations at low [CO_2_] (107 μmol CO_2_·Kg^−1^ SW). However, the abundance of the photorespiratory metabolites glycerate, glycerate 3-P and succinate semialdehyde (GABA shunt) were higher in DBW leaves than in SBV leaves (Fig. 4b, Table S7). The increase in succinate semialdehyde abundance under low [CO_2_] in DBW could represent a potential stress response as the GABA shunt may help prevent the accumulation of reactive oxygen intermediates^31,32,44,45^. SBV plants growing under low [CO_2_] (107 μmol CO_2_·Kg^−1^ SW) had higher abundance of proline and the sugar alcohol myo-inositol (Fig. 4b, Table S7), the latter are known to generate protein stabilizing osmolytes, such as di-myo-inositol phosphate^25^ that may help protect this population from heat stress^25^.

### South Bay comparison across CO_2_ treatments

**Figure 5:**
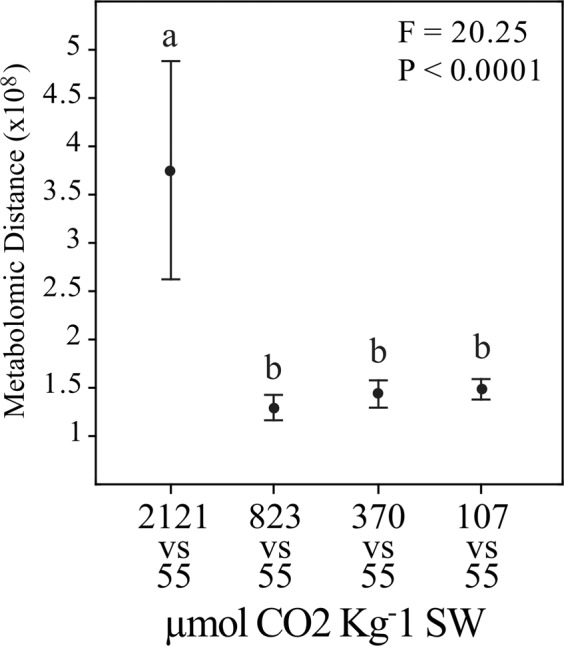
South Bay metabolomic distances (Mean ± Confidence Intervals 95%) between plants growing at ambient CO_2_ (55 μmol CO_2_·kg^−1^SW) and plants higher CO_2_ concentrations (2121, 823, 370, and 107 μmol CO_2_·kg^−1^SW). Fisher’s *F*-statistic and *p* value of the one-way ANOVA comparing the distances are indicated.

The SBV plants were grown across a CO_2_ concentration gradient as part of a related experiment^10^, enabling us to examine their metabolomic responses to different [CO_2_] in some detail. From the over 5,000 detected metabolic features, 455 (9%) increased with [CO_2_] and 408 (8.1%) decreased in response to [CO_2_]. To date, we identified 131 of the 863 responsive metabolites. Experimental CO_2_ enrichment elevated the concentration of intermediates associated with carbon fixation and amino acid synthesis, as well as sucrose, the latter which is consistent with our prior experimental findings^11,16^. PERMANOVA of the metabolomic fingerprints of SBV plants showed clear statistical differences between CO_2_ treatments (*p* < 0.05, Table S8). PCA distinctly clustered SBV plants growing at the highest CO_2_ treatment (2121 μmol CO_2_·Kg^−1^ SW) from the rest along the first principal component axis (PC1), which explains over 30% of the total variability (Fig. 3B), suggesting that plants growing at the highest CO_2_ levels experienced the largest metabolic changes. On the other hand, plants growing at ambient CO_2_ were separated from the other CO_2_ treatments along the second principal component axis (PC2). This trend was also observed with the Euclidian distances between the metabolomes of plants growing at higher CO_2_ levels (2121, 823, 370, and 107 CO_2_·Kg^−1^ SW) vs. plants growing at ambient [CO_2_]. The most dramatic overall metabolome change was detected between the highest (2121 μmol CO_2_·Kg^−1^ SW) and the ambient (55 μmol CO_2_·Kg^−1^ SW) [CO_2_] (Fig. 5), consistent with our PCA (Fig. 3B) and with the negative log-linear relationship between [CO_2_] and whole plant performance (Fig. 2). SBV plants grown under high [CO_2_] had higher abundance of glutamate (Fig. 4c, Table S9) which is involved in N assimilation^46^. In addition, enhancement of gluconate 6-P in SBV plants under high [CO_2_] (Fig. 4c, Table S9) suggests activation of the pentose phosphate pathway^47^ that leads to the synthesis of aromatic amino acids such as phenylalanine; another critical compound in protein synthesis as well as the formation of cell wall components, including lignin^48^. Univariate analyses showed that SBV plants exposed to ambient [CO_2_] produced higher abundance of TCA cycle intermediates (Fig. 4c) such as citrate, α-ketoglutarate, pyruvate, and GABA (Table S9). However, we found no differences in leaf dark respiration rates across different [CO_2_] treatments or between eelgrass populations when exposed to different temperatures (Fig. S2), suggesting that the increases of TCA Cycle metabolites in plants under ambient CO_2_ may have been diverted to other metabolic pathways (e.g. Shikimate) rather than enhancing respiratory ATP production. Although depriving the plant of potential energy for growth, such diversion leads to the synthesis of secondary compounds with diverse physiological roles, such as cell signaling, production of stress-related compounds and the formation of metabolites associated with the biosynthesis of polyphenols^49^. Using a small range of CO_2_ conditions, *Cymodocea nodosa* revealed up-regulation of genes coding for respiratory metabolism, increasing energetic demand for biosynthesis and stress-related processes under similar ambient [CO_2_] (pH 7.8/[CO_2_] 43 μmol Kg^−1^ SW)^50^. An accurate quantification of this diversion of respiratory intermediates to different pathways may serve as a good proxy for calculating the energetic cost of the physiological stress response to growth and reproductive output.

### Dumas Bay comparison between high and low CO_2_

For DBW plants, a low number of replicates in some CO_2_ treatments did not enable metabolomics analyses across all treatments. However, PERMANOVA did not find overall metabolomic differences between plants growing at low [CO_2_] (107 μmol Kg^−1^ SW) and plants under high [CO_2_] (823 μmol Kg^−1^ SW) (Table S8). The two clustered in different regions along PC1 (Fig. 3C) but according to individual ANOVAs of the metabolomic fingerprints detected significant changes between CO_2_ treatments (Tables S9 and S10). Under low [CO_2_] (107 μmol CO_2_·Kg^−1^ SW), DBW plants contained higher levels of α-ketoglutarate, succinate, glutamate and glycerate 3-P (Fig. 4d, Table S10) suggesting activation of the GABA shunt as a way to mitigate stress^36^. On the other hand, high [CO_2_] (823 μmol CO_2_·Kg^−1^ SW) stimulated the abundance of shikimate and proline (Fig. 4d, Table S10) the latter may have increased the thermal tolerance of this population.

## Conclusion

Our results revealed that eelgrass populations from two very different thermal environments both exhibited enhanced photosynthetic energy capture, sucrose formation and growth under CO_2_ enrichment that increased thermal tolerance, potentially counteracting some climate warming impacts on this foundational species. Both populations exhibited positive whole plant responses to CO_2_ in terms of leaf sucrose, leaf growth, and shoot numbers, but metabolite profiles hint at important genetic differences between these populations. Metabolomic analyses suggest that stress diverts the flow of photosynthates from growth and energy (ATP) production to non-anabolic intermediates that may help (i) elucidate important mechanisms responsible for stress tolerance and (ii) quantify the energetic cost of the stress response.

Although the differences in metabolite pools observed here in response to different [CO_2_] point to shifts in the activities of metabolic pathways leading to whole plant responses to potential climate forcing, we note that metabolite pool sizes alone are insufficient to fully understand the physiological basis for whole-plant responses to climate-driven environmental change. In addition to making more detailed analyses of metabolite change over time, analyses of changes in the proteome and transcriptome will be necessary to fully understand key genomic functions and metabolic pathways, and those analyses are currently under way. However, the metabolite profiles generated here, in combination with analysis of whole-plant performance, provide a force multiplier for translating ‘omic’ approaches into a predictive understanding of the physiological response of seagrasses to an increasingly hot and sour sea, and the potential for populations to adapt to new environments. Such knowledge will help predict earth system interactions in the context of global cycles and help inform best practices for seagrass restoration.

## Methods

### Plant collection

Eelgrass shoots with at least 5 rhizomatous internodes and roots were collected by hand from populations in (i) South Bay, Virginia, near the southern limit of their distribution in the Western Atlantic that experience a large seasonal temperature range and (ii) Dumas Bay in southern Puget Sound, WA that are less vulnerable to summertime thermal stress (Fig. S1). Shoots were carefully uprooted by hand, washed free of all sediment and transported to the experimental growth facility at the Virginia Aquarium & Marine Science Center, Virginia Beach, VA. Shoots from Dumas Bay, WA were packed in paper towels moistened with seawater, shipped overnight to VA and immediately transplanted into the experimental facility.

### Experimental facility

Eelgrass shoots were grown in fully replicated aquaria using the climate change experimental facility at the Virginia Aquarium & Marine Science Center^11^. The experimental system consisted of 20 outdoor aquaria plumbed with running water (10 turnovers/day) pumped from the adjacent Owls Creek estuary (Fig S3). Shoots were transplanted into rectangular fiberglass-reinforced plastic containers (0.04 m^3^ volume, 0.075 m^2^ surface area) filled with clean intertidal sand and placed in each aquarium. CO_2_ concentrations were manipulated using beverage-grade CO_2_ delivered through pH-controlled CO_2_ bubblers to maintain aquaria at five CO_2_ concentrations ranging from ambient (55 μmol CO_2_ Kg^−1^ SW, pH ~8.0) to 2121 μmol CO_2_ Kg^−1^ SW (pH 6) that encompasses a 200-year projection for CO_2_ availability and yielded a 3-fold gradient in light-saturated photosynthesis for the duration of the experiment^10,51^.

### Whole plant performance

Shoot counts, size, growth, and sucrose content of leaf tissues were measured each month to track the temporal changes across the CO_2_ treatment range as previously described^11^.

### Metabolic profiling

#### Tissue collection, storage and processing

One leaf sample (2nd youngest leaf) was collected monthly at random from each plastic container (three plastic containers for SBV and one for DBW in every aquarium). Epiphytes were removed by gently scraping each leaf with a clean razor blade, followed by a brief rinse in 0.2 μm-filtered seawater. The clean leaves were patted dry with a tissue, flash frozen in liquid nitrogen and stored at −80 °C. Samples collected in May 2014 used for the metabolomics analyses performed here were lyophilized for at least 48 h and powdered using a ball mill. The powdered samples were incubated in methanol/deionized water (4/1 v/v) at 10 °C on an orbital shaker (1 h), followed by gentle sonication for 2 min using a Branson ultrasonic cleaner (40 kHz). The extracts were centrifuged and the supernatants transferred to pre-combusted (450 °C for 8 h) amber glass vials for metabolite analysis. Three solvent-only vials were prepared using only methanol/deionized water (no plant material) processed as above.

#### GC-MS analyses of plant tissues

50 μL of eelgrass extract from each sample was dried and subsequently derivatized in two different steps^52^. First, compounds were derivatized to a trimethylsilyl ester form using methoxyamine in pyridine solution (30 mg/mL). 20 μL of methoxyamine solution was added to each dried extract and samples were incubated at 37 °C for 90 min in a Thermomixer operating at 1,200 rpm. Later, amine, carboxyl and hydroxyl groups were derivatized using 80 μL of MSTFA (N-Methyl-N-(trimethylsilyl) trifluoroacetamide), subsequently incubated at 37 °C for 30 min at 1,200 rpm. After derivatization, all extracts were vortexed for 10 s and centrifuged at 2,750 × g for 5 minutes and supernatants were used for GC-MS analyses.

GC-MS analyses were performed using an Agilent GC 7890A equipped with an HP-5MS column (30 m × 0.25 mm × 0.25 μm; Agilent Technologies) coupled to a MSD 5975C mass spectrometer (Agilent Technologies, Santa Clara, CA). The injection port temperature was 250 °C. Injection volume was set at 10 μL and split-less. The column was maintained at 60 °C for 1 min and then increased at a rate of 10 °C min^−1^ to 325 °C during the following 26.5 min and held for 10 min. Experimental blanks from the solvent-only vials were injected every 15 samples and a mixture of fatty acid methyl esters (FAMEs; C8-C28) was analyzed at the beginning of the sequence to calculate retention indices (RI).

Chromatograms were deconvoluted and aligned according to the RI that were calculated from the FAMEs mixture run at the beginning of the sequence. Metabolite identification was conducted by matching MS spectra and RI to an updated version of FiehnLib^53^. Assigned metabolites were subsequently validated using fragmentation spectra from NIST14 GC-MS library. Parameters used in the Metabolite detector are shown in Table S11. Metabolite matching information in GC-MS is shown in Table S12 and more details as previously described^52^.

#### LC-MS analyses of plant tissues

LC-MS analyses were performed using a Vanquish ultra-high pressure liquid chromatography system (UHPLC) coupled to an LTQ Orbitrap Velos mass spectrometer equipped with heated electrospray ionization (HESI) source (Thermo Fisher Scientific, Waltham, Massachusetts, USA). Chromatography was performed with a Hypersil gold C18 reversed-phase column (150 × 2.1 mm, 3 μ particle size; Thermo Scientific, Waltham, Massachusetts, USA) operating at 30° C. Mobile phases consisted of 0.1% formic acid in water (A) and 0.1% formic acid in acetonitrile/water (90:10) (B). The injection volume was 5 μL and flow rate was constant at 0.3 mL min^−1^. The elution gradient started at 90% A (10% B) constant for 5 min and then linearly changed to 10% A (90% B) during the following 15 min. Those conditions were held for 2 min before the initial conditions were recovered during the subsequent 2 min and the column was washed and stabilized for 11 min. All samples were injected in both negative (−) and positive (+) ionization modes. The MS operated at a resolution of 60,000 in Fourier Transform Mass Spectrometry (FTMS) full-scan mode measuring a mass range of 50 to1000 m/z. Experimental blanks from the solvent-only vials were injected every 15 samples. Details on the MS was previously described^54^.

LC-MS negative and positive chromatograms were separately processed with MZmine 2.26^55^. Chromatograms were baseline corrected, deconvoluted, aligned and metabolic features were assigned to metabolites according to retention time (RT) and exact mass of standard compounds included in our in-house library (second level identification according to Sumner *et al*.^56,57^). The parameters used for the extraction of the metabolic fingerprints are given in Table S13. Metabolite matching information in LC-MS is shown in Table S14.

### Statistical analysis

#### Whole plant performance

Within-aquarium replicate measures of each performance property were combined each month to generate statistically independent means for each aquarium (without error), resulting in statistically independent replicate measurements for each CO_2_ treatment each month. Consequently, statistical significance of treatment effects was determined using a repeated-measures ANCOVA implemented in the mixed model analysis of the linear mixed model component of IBM SPSS Statistics 22 using population and month as the fixed factors (within subjects) and log CO_2_ as the covariate (between subjects). The time series observations were treated as repeated subjects for each measured parameter. All error terms were expressed as standard errors unless otherwise noted.

#### Metabolomics

The final metabolomic dataset was composed of two categorical factors (Population and CO_2_ treatment) and 5757 continuous variables (metabolomic features), including 133 metabolites identified by our LC-MS and GC-MS compound libraries. Full factorial PERMANOVAs (Population + CO_2_ + Population × CO_2_) were performed to test for overall metabolomic differences between populations and CO_2_ levels. Both populations were exposed to all five [CO_2_] levels however for the metabolomics analyses the DBW population had a low number of replicates in some CO_2_ treatments constraining the metabolomics analyses across all treatments. Therefore, we only used two levels of CO_2_ for both populations (823 and 107 μmol CO_2_·Kg^−1^ SW) to be consistent with both populations for the full PERMANOVA model. Additional PERMANOVAs were performed to test for overall differences for CO_2_ treatments within each eelgrass population. All PERMANOVAs were computed using the Euclidean distance and 10,000 permutations. In addition, one-way ANOVAs were performed on individual  metabolic features contrasting (i) DBW vs. SBV plants at high [CO_2_] (823 μmol CO_2_ kg^−1^ SW), (ii) DBW vs. SBV plants at low [CO_2_] (107 μmol CO_2_ kg^−1^ SW), (iii) highest [CO_2_] (2121 μmol CO_2_ kgSW^−1^) vs. ambient [CO_2_] (55 μmol CO_2_ kgSW^−1^) for SBV plants, and (iv) high [CO_2_] (823 μmol CO_2_ kgSW^−1^) vs. low [CO_2_] (107 μmol CO_2_ kgSW^−1^) for DBW plants.

The datasets (SBV + DBW, SBV alone, and DBW alone) were subsequently subjected to principal component analysis (PCA) to visualize the overall metabolomic variability of the study cases along the two axes explaining most variability (principal component (PC) 1 and PC2).

The Euclidean distances (metabolomic distances) between plants growing at ambient CO_2_ (55 μmol CO_2_· kg^−1^SW) and plants at higher CO_2_ concentrations (2121, 823, 370, and 107 µmol CO_2_ kg^−1^SW) were calculated using the entire metabolomic fingerprints. Those distances were posteriorly submitted to one-way ANOVAs followed by Tukey HSD post-hoc test to understand which [CO_2_] had a higher response in comparison to ambient [CO_2_]. Pearson correlation coefficients (*r*) between CO_2_ gradient and individual metabolic features for SBV plants were calculated.

All statistical analyses for metabolomics were performed in R version 3.6.1^58^. PERMANOVAs were performed using the *adonis* function from the package “vegan”^59^. One-way ANOVAs and Euclidian distances were calculated with the functions *aov* and *dist*, respectively, included in the package “stats”^58^. Tuskey HSD post hoc tests were conducted using the HSD.test function from the agricolae package^60^. All PCAs were performed using the function *PCA* found in 2323 “FactoMineR” package^61^. For PCAs, missing data were previously imputed using the function imputePCA from “missMDA” package^62^.

## Supplementary information

Supplementary Information.

## Data Availability

Whole-plant metabolic data are available from BCO-DMO (Principal Investigator Richard Zimmerman, Project: Impact of Climate Warming and Ocean Carbonation on Eelgrass (*Zostera marina* L.). Metabolomics data are also available in the Supporting Information for this manuscript.
