# Supplementary material for: Metabolic Profiling Reveals Biochemical Pathways Responsible for Eelgrass Response to Elevated CO2 and Temperature
Source: Sci Rep. 2020 Mar 13;10:4693. doi: 10.1038/s41598-020-61684-x (PMC7070064; doi:10.1038/s41598-020-61684-x)
Supplement: Supplementary file 1 — Supplementary Information. [file 41598_2020_61684_MOESM1_ESM.docx]

**Metabolic Profiling Reveals Biochemical Pathways Responsible for Eelgrass Response to Elevated CO_2_ and Temperature**

Carmen C. Zayas-Santiago^,1^, Albert Rivas-Ubach^2^, Li-Jung Kuo^3^, Nicholas D. Ward^3-4^, Richard C. Zimmerman^1^

^1^Department of Ocean, Earth & Atmospheric Sciences, Old Dominion University, Norfolk VA 23429, USA

^2^Pacific Northwest National Laboratory, P.O. Box 999, Richland WA 99352, USA

^3^Marine Sciences Laboratory, Pacific Northwest National Laboratory, 1529 W. Sequin Bay Rd., Sequim WA 98382, USA

^4^School of Oceanography, University of Washington, Seattle, WA 98105, USA

Supplemental Information

# Figures

**Figure S1**. Daily average water temperature during 2013 and 2014 at Owl’s Creek, VA (red circles)^10^ and at the NOAA buoy closest to Dumas Bay (Station 9446484) (blue circles) during 2013-2014. WA Temperature Data [http](file:///C:\RCZ\Papers\Seagrass%20Co2%20metabolome\Version%208\http)[://tidesandcurrents.noaa.gov/physocean.html](http://tidesandcurrents.noaa.gov/physocean.html). The black line indicates the 25° C threshold for the onset of thermal stress in eelgrass.

**Figure S2** Leaf respiration rates vs. temperature coming from high (823 *µ*mol CO_2_ kg^-1^ SW ) and ambient (55 *µ*mol CO_2_ kg^-1^ SW ) CO_2_ treatments. Measured in the oxygen electrode chamber at ambient CO_2_. Error bars represent ±1 SE.


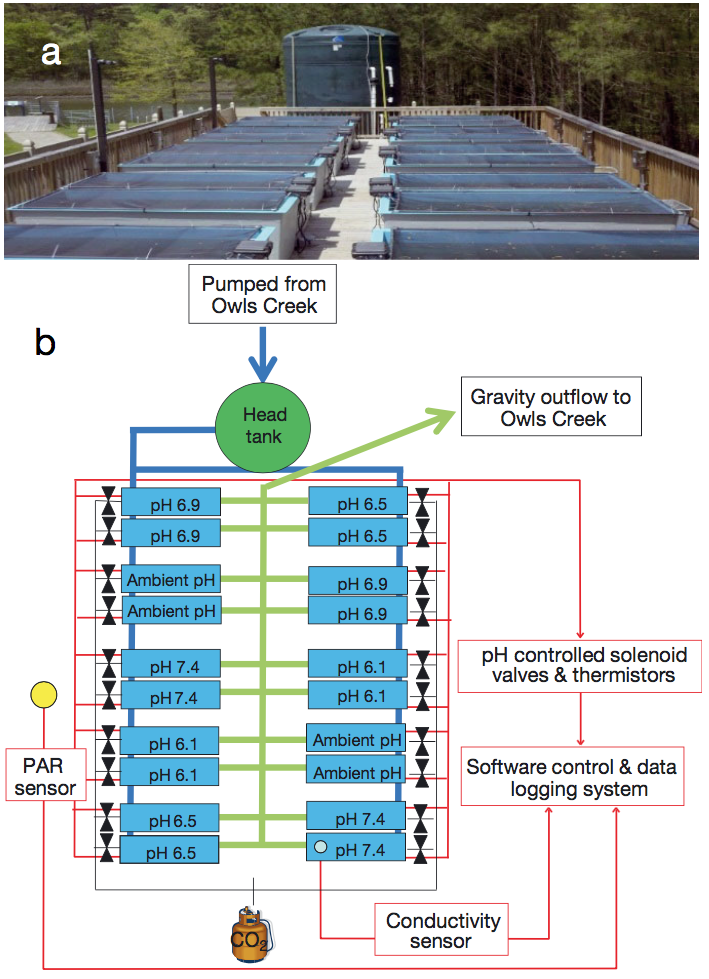


# Tables

**Figure S3** (a) Experimental climate change facility at the Virginia Aquarium & Marine Science Center showing the 20 fiberglass tanks (3 m^3^ each), CO_2_ control units mounted on each aquarium, and the large head tank. (b) Schematic diagram of the experimental system, illustrat- ing the experimental aquaria (blue rectangles), water inflow (blue lines) and outflow (green lines), CO_2_ supply delivery system (tank black lines) with pH-controlled valves (black hourglass symbols), and environmental monitoring system (red lines and rectangles). Reproduced here from ^11^ (Figure 1).

## Whole Plant Performance Stats

**Table S1.** Percent Change in Plant Size ANCOVA table for Type III tests of fixed effects using the mixed linear model routine implemented in SPSS. Month and population were treated as as fixed factors with log [CO_2_] as the covariate.

| **Source** | ***df* Numerator** | ***df* Denominator** | ***F*** | ***p*** |
| --- | --- | --- | --- | --- |
| Intercept | 1 | 60.71 | 6.50 | 0.013 |
| Population | 2 | 51.22 | 4.81 | 0.012 |
| Month | 12 | 27.61 | 2.28 | 0.036 |
| log[CO_2_] | 1 | 96.3 | 22.87 | <0.001 |
| Population * log[CO_2_] | 2 | 51.60 | 7.11 | 0.002 |
| Month * log[CO_2_] | 12 | 29.73 | 1.32 | 0.255 |
| Population * Month * log[CO2] | 11 | 23.82 | 1.30 | 0.281 |

**Table S2**. Percent Survival ANCOVA table for Type III tests of fixed effects using the mixed linear model routine implemented in SPSS. Month and population were treated as fixed factors with log [CO_2_] as the covariate.

| **Source** | ***df* Numerator** | ***df* Denominator** | ***F*** | ***p*** |
| --- | --- | --- | --- | --- |
| Intercept | 1 | 94.08 | 6.85 | 0.010 |
| Population | 1 | 3 x10^29^ | .000 | 1.000 |
| Month | 12 | 11.21 | 13.36 | <0.001 |
| Log [CO_2_] | 1 | 111.36 | 129.64 | 0.001 |
| Population * log [CO_2_] | 1 | 45.75 | 4.47 | 0.040 |
| Month * log [CO_2_] | 12 | 29.47 | 41.65 | <0.001 |
| Month * Population * log [CO_2_] | 11 | 9.95 | 4.10 | 0.017 |

**Table S3**. Percent Growth Rate ANCOVA table for Type III tests of fixed effects using the mixed linear model routine implemented in SPSS. Month and population were treated as fixed factors with log [CO_2_] as the covariate.

| **Source** | ***df* Numerator** | ***df* Denominator** | ***F*** | ***p*** |
| --- | --- | --- | --- | --- |
| Intercept | 1 | 135.44 | 191.96 | <0.001 |
| Population | 1 | 86.58 | 6.21 | 0.015 |
| Month | 12 | 23.20 | 8.08 | <0.001 |
| log [CO_2_] | 1 | 141.22 | 1.34 | 0.249 |
| Population * log [CO_2_] | 1 | 92.56 | 8.47 | 0.005 |
| Month* log [CO_2_] | 12 | 26.15 | 3.09 | 0.008 |
| Month * Population * log [CO_2_] | 11 | 27.16 | 3.10 | 0.008 |

**Table S4.** Sucrose (µmol g^-1^ DW) ANCOVA table for Type III tests of fixed effects using the mixed linear model routine implemented in SPSS. Month and population were treated as fixed factors with log [CO_2_] as the covariate.

| **Source** | ***df* Numerator** | ***df* Denominator** | ***F*** | ***p*** |
| --- | --- | --- | --- | --- |
| Intercept | 1 | 145.71 | 2.02 | 0.157 |
| Population | 1 | 130.74 | .127 | 0.722 |
| Month | 11 | 49.80 | 3.51 | 0.001 |
| log [CO_2_] | 1 | 154.60 | 143.74 | <0.001 |
| Population * log [CO_2_] | 1 | 143.59 | 5.09 | 0.025 |
| Month * log [CO_2_] | 11 | 55.23 | 5.15 | <0.001 |
| Month * Population * log [CO_2_] | 11 | 22.14 | 23.26 | <0.001 |

## Metabolomics Statistics

**Table S5.** Summary Factorial PERMANOVA

|  | **Source** | ***df*** | **Sum of Squares** | **Mean Square** | ***F*** | ***p*** |
| --- | --- | --- | --- | --- | --- | --- |
| All populations, All log [CO_2_] | log [CO_2_] | 1 | 1.7E + 17 | 1.7E+17 | 6.46 | <0.001 |
|  | Population | 1 | 1.5E17 | 1.5E+17 | 5.41 | <0.001 |
|  | log[CO_2_] x Population | 1 | 6.5E+16 | 6.5E+16 | 2.41 | 0.077 |

**Table S6.** ANOVA summary table for differences in relative abundance (MS peak area) of identified leaf metabolites for the high [CO_2_] (823 *µ*mol CO_2_ kg^-1^ SW) treatment.

| **Metabolite** | **KEGG ID** | **Dumas Bay WA**  **Mean ± SE MS Peak Area** | **South Bay VA**  **Mean ± SE MS Peak Area** | ***F*** | ***p*** | **Higher concentration** |
| --- | --- | --- | --- | --- | --- | --- |
| L.Serine | C00716 | 16.83E+04 ± 70.83E+02 | 8.60E+04 ± 30.00E+02 | 141.81 | < 0.01 | Dumas Bay, WA |
| Guanosine | C00387 | 3.00E+04 ± 24.28E+02 | 48.08E+02 ± 6.92E+02 | 132.18 | < 0.01 | Dumas Bay, WA |
| S.1.Phenylethanol | C07112 | 74.09E+04 ± 3.37E+04 | 23.77E+04 ± 2.94E+04 | 126.42 | < 0.01 | Dumas Bay, WA |
| Cytosine | C00380 | 41.57E+04 ± 3.66E+04 | 7.83E+04 ± 40.62E+02 | 118.45 | < 0.01 | Dumas Bay, WA |
| Guanine | C00242 | 54.94E+04 ± 2.76E+04 | 17.85E+04 ± 2.61E+04 | 92.37 | < 0.01 | Dumas Bay, WA |
| 4.Hydroxy.L.Proline | C01157 | 10.72E+04 ± 71.02E+02 | 5.26E+04 ± 34.93E+02 | 56.91 | < 0.01 | Dumas Bay, WA |
| Uracil | C00106 | 16.81E+04 ± 91.72E+02 | 7.61E+04 ± 1.00E+04 | 42.35 | < 0.01 | Dumas Bay, WA |
| Sugars.Alcohol.Hexoses |  | 96.95E+02 ± 13.34E+02 | 21.64E+02 ± 4.18E+02 | 38.09 | < 0.01 | Dumas Bay, WA |
| L.Proline | C16435 | 79.17E+06 ± 2.13E+06 | 61.69E+06 ± 1.88E+06 | 37.67 | < 0.01 | Dumas Bay, WA |
| Nicotinamide | C00153 | 1.11E+06 ± 6.34E+04 | 69.68E+04 ± 4.33E+04 | 31.87 | < 0.01 | Dumas Bay, WA |
| D.Arabinose | C00216 | 78.30E+04 ± 62.38E+02 | 27.09E+04 ± 8.16E+04 | 28.08 | < 0.01 | Dumas Bay, WA |
| Shikimate | C00493 | 66.48E+04 ± 7.94E+04 | 32.14E+04 ± 2.15E+04 | 23.31 | < 0.01 | Dumas Bay, WA |
| Glyceraldehyde | C02154 | 40.84E+04 ± 5.64E+04 | 10.28E+04 ± 3.79E+04 | 22.04 | 0.01 | Dumas Bay, WA |
| 3.Dehydroshikimate | C02637 | 73.98E+02 ± 16.20E+02 | 14.08E+02 ± 4.92E+02 | 16.48 | 0.01 | Dumas Bay, WA |
| Pyridoxine | C00314 | 16.31E+04 ± 1.99E+04 | 7.91E+04 ± 1.13E+04 | 15.39 | 0.01 | Dumas Bay, WA |
| 5.Methylcytosine.Hydrocloride | C02376 | 5.16E+04 ± 89.69E+02 | 2.06E+04 ± 21.03E+02 | 15.35 | 0.01 | Dumas Bay, WA |
| 4.Acetamidobutanoate | C02946 | 11.80E+04 ± 2.17E+04 | 4.35E+04 ± 62.86E+02 | 14.37 | 0.01 | Dumas Bay, WA |
| Galactitol | C01697 | 84.07E+02 ± 20.09E+02 | 31.34E+02 ± 1.59E+02 | 9.72 | 0.03 | Dumas Bay, WA |
| 5.Methylthioadenosine | C00170 | 4.48E+04 ± 22.82E+02 | 2.57E+04 ± 51.65E+02 | 8.87 | 0.03 | Dumas Bay, WA |
| Deoxy-Hexoses |  | 60.58E+02 ± 13.94E+02 | 21.70E+02 ± 5.35E+02 | 8.59 | 0.03 | Dumas Bay, WA |
| Adenine | C00147 | 7.66E+06 ± 56.81E+04 | 3.66E+06 ± 1.14E+06 | 7.80 | 0.04 | Dumas Bay, WA |
| Hypoxanthine | C00262 | 15.34E+04 ± 6.12E+04 | 1.39E+04 ± 16.83E+02 | 7.41 | 0.04 | Dumas Bay, WA |
| Naringenin | C00509 | 22.80E+02 ± 9.49E+02 | 1.40E+02 ± 4.21E+00 | 7.26 | 0.04 | Dumas Bay, WA |
| Thymine | C00178 | 7.87E+04 ± 76.41E+02 | 4.30E+04 ± 1.06E+04 | 6.46 | 0.05 | Dumas Bay, WA |
| Arabitol | C01904 | 56.96E+04 ± 5.56E+04 | 36.96E+04 ± 5.39E+04 | 6.42 | 0.05 | Dumas Bay, WA |
| Rs.Mevalonic.Acid | C00418 | 51.14E+02 ± 4.67E+02 | 25.29E+02 ± 8.45E+02 | 5.80 | 0.06 | Dumas Bay, WA |
| Eriodictyol | C05631 | 11.96E+02 ± 5.42E+02 | 1.30E+02 ± 14.68E+00 | 5.53 | 0.07 | Dumas Bay, WA |
| D.Pantothenic.Acid | C00864 | 10.41E+04 ± 3.41E+04 | 3.71E+04 ± 27.40E+02 | 5.45 | 0.07 | Dumas Bay, WA |
| Diethanolamine | C06772 | 6.78E+04 ± 3.31E+04 | 33.73E+02 ± 7.08E+02 | 5.39 | 0.07 | Dumas Bay, WA |
| Pyruvate | C00022 | 5.69E+04 ± 20.32E+02 | 3.14E+04 ± 96.51E+02 | 4.91 | 0.08 | Dumas Bay, WA |
| L.Threonine | C00188 | 18.64E+04 ± 3.22E+04 | 11.14E+04 ± 2.03E+04 | 4.31 | 0.09 | Dumas Bay, WA |
| L.Pipecolic.Acid | C00408 | 1.49E+06 ± 37.59E+04 | 83.29E+04 ± 8.45E+04 | 3.95 | 0.10 | Dumas Bay, WA |
| Creatine | C00300 | 81.76E+04 ± 47.44E+04 | 4.86E+04 ± 67.21E+02 | 3.75 | 0.11 | Dumas Bay, WA |
| 2.Aminophenol | C01987 | 72.94E+04 ± 5.98E+04 | 59.72E+04 ± 4.30E+04 | 3.43 | 0.12 | Dumas Bay, WA |
| Palmitic.Acid | C00249 | 2.45E+06 ± 4.92E+04 | 1.93E+06 ± 23.60E+04 | 3.36 | 0.13 | Dumas Bay, WA |
| D.3.Phosphoglyceric.Acid | C00597 | 95.48E+02 ± 53.36E+02 | 16.63E+02 ± 3.09E+02 | 3.10 | 0.14 | Dumas Bay, WA |
| 3.Amino.5.Hydroxybenzoic.Acid | C12107 | 3.48E+04 ± 18.80E+02 | 2.85E+04 ± 27.33E+02 | 3.07 | 0.14 | Dumas Bay, WA |
| Monoshaccharides Hexoses |  | 1.86E+06 ± 77.52E+04 | 69.53E+04 ± 13.23E+04 | 3.04 | 0.14 | Dumas Bay, WA |
| L.Valine | C00183 | 13.72E+06 ± 7.03E+06 | 3.67E+06 ± 72.09E+04 | 2.86 | 0.15 | Dumas Bay, WA |
| Acetoacetate | C00164 | 6.94E+04 ± 17.89E+02 | 5.51E+04 ± 70.97E+02 | 2.80 | 0.15 | Dumas Bay, WA |
| D.Mannose | C00159 | 1.85E+06 ± 72.02E+04 | 85.30E+04 ± 8.80E+04 | 2.67 | 0.16 | Dumas Bay, WA |
| L.Arginine | C00062 | 1.00E+04 ± 24.94E+02 | 50.09E+02 ± 17.13E+02 | 2.79 | 0.17 | Dumas Bay, WA |
| 4.Guanidinobutanoate | C01035 | 22.37E+04 ± 11.96E+04 | 6.23E+04 ± 1.60E+04 | 2.51 | 0.17 | Dumas Bay, WA |
| Glutaric.Acid | C00489 | 11.32E+04 ± 2.64E+04 | 6.64E+04 ± 1.85E+04 | 2.26 | 0.19 | Dumas Bay, WA |
| Mandelic.Acid | C01984 | 2.10E+04 ± 85.77E+02 | 88.28E+02 ± 34.07E+02 | 2.18 | 0.20 | Dumas Bay, WA |
| S.Malate | C00711 | 5.36E+06 ± 2.48E+06 | 2.08E+06 ± 68.49E+04 | 2.16 | 0.20 | Dumas Bay, WA |
| Succinate.Semialdehyde | C00232 | 1.16E+04 ± 30.63E+02 | 58.28E+02 ± 26.05E+02 | 2.09 | 0.21 | Dumas Bay, WA |
| D.Lyxosylamine |  | 1.91E+06 ± 31.60E+04 | 1.43E+06 ± 17.49E+04 | 2.03 | 0.21 | Dumas Bay, WA |
| Histamine | C00388 | 4.76E+04 ± 59.27E+02 | 3.89E+04 ± 31.78E+02 | 1.96 | 0.22 | Dumas Bay, WA |
| Pyruvic.Aldehyde | C00546 | 20.55E+04 ± 9.51E+04 | 9.25E+04 ± 2.49E+04 | 1.77 | 0.24 | Dumas Bay, WA |
| Gallic.Acid | C01424 | 69.77E+02 ± 8.10E+02 | 44.02E+02 ± 16.46E+02 | 1.56 | 0.27 | Dumas Bay, WA |
| Fumarate | C00122 | 15.47E+04 ± 5.17E+04 | 9.48E+04 ± 2.38E+04 | 1.35 | 0.30 | Dumas Bay, WA |
| Sucrose | C00089 | 1.26E+08 ± 6.37E+06 | 1.11E+08 ± 10.27E+06 | 1.30 | 0.31 | Dumas Bay, WA |
| D.Gulonic.Acid.Gama.Lactone | C01040 | 3.69E+04 ± 1.04E+04 | 2.25E+04 ± 80.38E+02 | 1.25 | 0.31 | Dumas Bay, WA |
| 4.Hydroxy.L.Phenylglycine.Pyridoxal | CA1445 | 18.83E+04 ± 7.99E+04 | 10.82E+04 ± 2.20E+04 | 1.25 | 0.31 | Dumas Bay, WA |
| Creatinine | C00791 | 11.50E+04 ± 5.92E+04 | 6.34E+04 ± 57.68E+02 | 1.07 | 0.35 | Dumas Bay, WA |
| L.Alanine | C00041 | 60.19E+04 ± 4.15E+04 | 47.35E+04 ± 10.33E+04 | 1.02 | 0.36 | Dumas Bay, WA |
| 3.Methoxytyramine | C05587 | 5.19E+04 ± 9.72E+02 | 4.49E+04 ± 57.89E+02 | 1.02 | 0.36 | Dumas Bay, WA |
| Phloroglucinol | C02183 | 7.62E+06 ± 1.29E+06 | 5.94E+06 ± 1.20E+06 | 0.90 | 0.39 | Dumas Bay, WA |
| Leucine | C16439 | 54.55E+04 ± 16.48E+04 | 40.77E+04 ± 5.74E+04 | 0.80 | 0.41 | Dumas Bay, WA |
| Urocanate | C00785 | 3.73E+04 ± 34.12E+02 | 3.46E+04 ± 11.16E+02 | 0.76 | 0.42 | Dumas Bay, WA |
| Alpha.Aminoadipate | C00956 | 10.57E+04 ± 89.31E+02 | 9.17E+04 ± 1.28E+04 | 0.68 | 0.45 | Dumas Bay, WA |
| Adenosine.5.Monophosphate | C00020 | 12.59E+04 ± 2.09E+04 | 10.92E+04 ± 93.03E+02 | 0.65 | 0.46 | Dumas Bay, WA |
| Hexoses.Phosphate |  | 4.67E+04 ± 2.50E+04 | 2.76E+04 ± 1.36E+04 | 0.52 | 0.50 | Dumas Bay, WA |
| Pyridoxamine | C00534 | 3.63E+04 ± 9.68E+02 | 3.08E+04 ± 64.99E+02 | 0.51 | 0.51 | Dumas Bay, WA |
| 4.Aminobutanoate (GABA) | C00334 | 35.74E+04 ± 1.62E+04 | 29.46E+04 ± 7.55E+04 | 0.48 | 0.52 | Dumas Bay, WA |
| 1.Methyladenine | C02216 | 3.62E+04 ± 93.22E+02 | 3.03E+04 ± 28.45E+02 | 0.48 | 0.52 | Dumas Bay, WA |
| Uridine | C00299 | 4.03E+04 ± 1.17E+04 | 3.27E+04 ± 46.63E+02 | 0.46 | 0.53 | Dumas Bay, WA |
| L.Sorbose | C00247 | 38.14E+06 ± 13.90E+06 | 29.10E+06 ± 5.62E+06 | 0.46 | 0.53 | Dumas Bay, WA |
| D.Malic.Acid | C00497 | 3.48E+06 ± 1.49E+06 | 2.63E+06 ± 18.19E+04 | 0.45 | 0.53 | Dumas Bay, WA |
| Luteolin | C01514 | 6.77E+06 ± 3.80E+06 | 4.90E+06 ± 1.30E+06 | 0.28 | 0.62 | Dumas Bay, WA |
| D-Fructose | C00095 | 56.19E+06 ± 22.86E+06 | 45.86E+06 ± 9.37E+06 | 0.22 | 0.66 | Dumas Bay, WA |
| D.Glucuronolactone | C00191 | 8.65E+04 ± 2.41E+04 | 7.27E+04 ± 2.42E+04 | 0.15 | 0.71 | Dumas Bay, WA |
| Disaccharides |  | 3.44E+06 ± 49.72E+04 | 2.95E+06 ± 1.04E+06 | 0.14 | 0.72 | Dumas Bay, WA |
| Phenylacetic.Acid | C07086 | 41.63E+02 ± 23.39E+02 | 33.73E+02 ± 13.24E+02 | 0.10 | 0.77 | Dumas Bay, WA |
| Nepsilon.Trimethyllysine | C03793 | 13.64E+02 ± 46.86E+00 | 12.63E+02 ± 2.76E+02 | 0.09 | 0.77 | Dumas Bay, WA |
| Succinate | C00042 | 6.40E+04 ± 38.40E+02 | 5.68E+04 ± 2.15E+04 | 0.08 | 0.79 | Dumas Bay, WA |
| L.Tyrosine | C01536 | 35.30E+04 ± 4.75E+04 | 33.45E+04 ± 5.96E+04 | 0.05 | 0.83 | Dumas Bay, WA |
| O.Succinyl.L.Homoserine | C01118 | 22.50E+04 ± 2.74E+04 | 22.07E+04 ± 3.78E+04 | 0.01 | 0.94 | Dumas Bay, WA |
| Caffeic.Acid | C01197 | 87.49E+04 ± 20.75E+04 | 87.38E+04 ± 9.72E+04 | 0.00 | 1.00 | Dumas Bay, WA |
|  |  |  |  |  |  |  |
| a.Ketoglutaric.Acid | C00026 | 1.09E+04 ± 29.89E+02 | 11.25E+04 ± 1.30E+04 | 57.84 | < 0.01 | South Bay, VA |
| N.Acetyl.D.Tryptophan | C03137 | 63.51E+02 ± 68.60E+00 | 1.75E+04 ± 23.78E+02 | 15.57 | 0.01 | South Bay, VA |
| 1.Aminocyclopropane.1.Carboxylate | C01234 | 2.29E+06 ± 12.23E+04 | 14.03E+06 ± 2.68E+06 | 13.66 | 0.01 | South Bay, VA |
| 2.6.Dihydroxypyridine | C03056 | 5.05E+04 ± 50.38E+02 | 8.77E+04 ± 88.83E+02 | 10.77 | 0.02 | South Bay, VA |
| Azelaic.Acid | C08261 | 34.55E+02 ± 8.16E+02 | 71.23E+02 ± 9.09E+02 | 8.29 | 0.03 | South Bay, VA |
| Galactonic.Acid | C00880 | 48.72E+04 ± 15.57E+04 | 90.02E+04 ± 6.29E+04 | 7.58 | 0.04 | South Bay, VA |
| 3.Amino.4.Hydroxybenzoic.Acid | C12115 | 4.23E+04 ± 96.25E+02 | 7.19E+04 ± 60.89E+02 | 7.53 | 0.04 | South Bay, VA |
| Nalpha.Acetyl.L.Lysine | C12989 | 3.50E+04 ± 84.95E+02 | 5.62E+04 ± 27.03E+02 | 7.42 | 0.04 | South Bay, VA |
| L.Isoleucine | C16434 | 1.97E+06 ± 55.89E+04 | 3.29E+06 ± 20.96E+04 | 6.20 | 0.06 | South Bay, VA |
| 4.Hydroxybenzaldehyde | C00633 | 41.61E+02 ± 1.88E+02 | 2.41E+04 ± 69.12E+02 | 5.92 | 0.06 | South Bay, VA |
| Rosmarinic.Acid | C01850 | 57.90E+04 ± 33.30E+04 | 1.79E+06 ± 34.93E+04 | 5.88 | 0.06 | South Bay, VA |
| Turanose | C19636 | 1.60E+06 ± 18.61E+04 | 2.44E+06 ± 26.06E+04 | 5.85 | 0.06 | South Bay, VA |
| N.Acetyl.L.Alanine | C01073 | 2.98E+04 ± 3.52E+02 | 3.66E+04 ± 27.71E+02 | 4.23 | 0.09 | South Bay, VA |
| N.Acetyl.Dl.Glutamic.Acid | C00624 | 2.06E+06 ± 34.20E+04 | 10.89E+06 ± 3.74E+06 | 3.98 | 0.10 | South Bay, VA |
| 5.Oxo.L.Proline | C01879 | 8.52E+06 ± 96.91E+04 | 24.63E+06 ± 6.86E+06 | 3.90 | 0.11 | South Bay, VA |
| L.Glutamine | C00303 | 14.20E+06 ± 2.30E+06 | 46.62E+06 ± 13.88E+06 | 3.84 | 0.11 | South Bay, VA |
| L.DOPA | C00355 | 52.57E+04 ± 23.02E+04 | 96.88E+04 ± 11.11E+04 | 3.61 | 0.12 | South Bay, VA |
| L.Asparagine | C16438 | 11.44E+04 ± 1.79E+04 | 32.37E+04 ± 10.81E+04 | 2.64 | 0.17 | South Bay, VA |
| Maleamate | C01596 | 3.00E+04 ± 51.71E+02 | 4.12E+04 ± 47.14E+02 | 2.52 | 0.17 | South Bay, VA |
| Salicylate | C00805 | 55.13E+02 ± 8.60E+02 | 4.59E+04 ± 2.23E+04 | 2.33 | 0.19 | South Bay, VA |
| Adenosine | C00212 | 23.58E+04 ± 13.25E+04 | 4.48E+06 ± 2.36E+06 | 2.31 | 0.19 | South Bay, VA |
| Citrate | C00158 | 2.11E+06 ± 87.25E+04 | 3.33E+06 ± 28.77E+04 | 2.30 | 0.19 | South Bay, VA |
| Formononetin | C00858 | 3.47E+02 ± 89.93E+00 | 13.71E+02 ± 6.21E+02 | 1.92 | 0.22 | South Bay, VA |
| Trigonelline | C01004 | 6.00E+06 ± 1.24E+06 | 8.89E+06 ± 1.57E+06 | 1.86 | 0.23 | South Bay, VA |
| Glutamic.Acid | C00025 | 3.90E+06 ± 45.34E+04 | 10.35E+06 ± 4.00E+06 | 1.85 | 0.23 | South Bay, VA |
| 3.2.Hydroxyphenyl.Propanoate | C01198 | 59.96E+02 ± 5.67E+02 | 72.65E+02 ± 7.10E+02 | 1.73 | 0.25 | South Bay, VA |
| D.Trehalose | C01083 | 1.79E+06 ± 25.93E+04 | 2.21E+06 ± 20.94E+04 | 1.61 | 0.26 | South Bay, VA |
| Salsolinol | C09642 | 3.05E+04 ± 17.30E+02 | 3.46E+04 ± 28.81E+02 | 1.22 | 0.32 | South Bay, VA |
| L.Phenylalanine | C02057 | 75.89E+04 ± 14.95E+04 | 1.39E+06 ± 52.67E+04 | 0.99 | 0.37 | South Bay, VA |
| Resorcinol.Monoacetate | C12064 | 88.44E+02 ± 29.12E+02 | 1.56E+04 ± 54.21E+02 | 0.96 | 0.37 | South Bay, VA |
| Sugars.Alcohol.Pentoses |  | 37.90E+02 ± 9.48E+02 | 46.92E+02 ± 6.38E+02 | 0.68 | 0.45 | South Bay, VA |
| 3.Hydroxykynurenine | C02794 | 8.44E+04 ± 3.04E+04 | 10.45E+04 ± 74.54E+02 | 0.56 | 0.49 | South Bay, VA |
| Myo.Inositol | C00137 | 47.75E+06 ± 5.72E+06 | 51.73E+06 ± 2.97E+06 | 0.45 | 0.53 | South Bay, VA |
| Glycerol.3.Phosphate | C00093 | 68.27E+04 ± 15.36E+04 | 88.96E+04 ± 26.73E+04 | 0.37 | 0.57 | South Bay, VA |
| Monosaccharides Pentoses |  | 12.64E+04 ± 1.84E+04 | 14.06E+04 ± 1.55E+04 | 0.35 | 0.58 | South Bay, VA |
| 3.Aminoisobutanoate | C05145 | 1.72E+04 ± 44.18E+02 | 2.19E+04 ± 59.68E+02 | 0.35 | 0.58 | South Bay, VA |
| 6.Phosphogluconic.Acid | C00345 | 7.33E+04 ± 2.22E+04 | 9.44E+04 ± 3.17E+04 | 0.25 | 0.64 | South Bay, VA |
| Fisetin | C10041 | 1.19E+08 ± 13.65E+06 | 1.25E+08 ± 6.21E+06 | 0.23 | 0.65 | South Bay, VA |
| Nicotinate.Picolinic.Acid | C00253 | 4.84E+04 ± 61.66E+02 | 5.37E+04 ± 93.59E+02 | 0.19 | 0.68 | South Bay, VA |
| N.Acetylglycine | CA1212 | 6.57E+04 ± 86.22E+02 | 7.16E+04 ± 99.85E+02 | 0.18 | 0.69 | South Bay, VA |
| Tyramine | C00483 | 4.08E+04 ± 95.01E+02 | 4.40E+04 ± 52.00E+02 | 0.10 | 0.76 | South Bay, VA |
| Quinoline | C06413 | 3.37E+04 ± 75.35E+02 | 3.62E+04 ± 41.50E+02 | 0.10 | 0.77 | South Bay, VA |
| Xylitol | C00379 | 10.81E+04 ± 2.55E+04 | 11.53E+04 ± 1.15E+04 | 0.08 | 0.79 | South Bay, VA |
| Aspartate | C00049 | 1.27E+06 ± 47.44E+04 | 1.42E+06 ± 31.77E+04 | 0.07 | 0.80 | South Bay, VA |
| 2.Hydroxypyridine | C02502 | 77.64E+04 ± 7.79E+04 | 82.81E+04 ± 16.38E+04 | 0.06 | 0.81 | South Bay, VA |
| Linoleic.Acid | C01595 | 18.06E+04 ± 5.69E+04 | 19.69E+04 ± 2.91E+04 | 0.06 | 0.81 | South Bay, VA |
| 6.Hydroxynicotinate | C01020 | 4.87E+04 ± 48.38E+02 | 5.03E+04 ± 43.36E+02 | 0.06 | 0.82 | South Bay, VA |
| 1.2.Phenylenediamine | C14402 | 4.87E+04 ± 1.21E+04 | 5.19E+04 ± 87.65E+02 | 0.05 | 0.83 | South Bay, VA |
| Glyceric.Acid | C00258 | 18.83E+04 ± 3.55E+04 | 19.27E+04 ± 1.51E+04 | 0.02 | 0.90 | South Bay, VA |
| Dehydroascorbate | C05422 | 56.00E+04 ± 26.29E+04 | 58.46E+04 ± 16.30E+04 | 0.01 | 0.94 | South Bay, VA |
| Amino-Sugars |  | 5.63E+04 ± 3.52E+04 | 5.86E+04 ± 38.16E+02 | 0.01 | 0.94 | South Bay, VA |

**Table S7.** ANOVA population comparison of identified leaf metabolites relative abundance (i.e. MS peak area) and standard error on low CO_2_ (107 µmol CO_2_ kgSW^-1^) treatment

| **Metabolite** | **KEGG ID** | **Dumas Bay WA**  **Mean ± SE MS Peak Area** | **South Bay VA**  **Mean ± SE MS Peak Area** | ***F*** | ***p*** | **Higher concentration** |
| --- | --- | --- | --- | --- | --- | --- |
| Glycerate 3P | C00597 | 7.63E+04 ± 78.57E+02 | 57.89E+02 ± 30.88E+02 | 87.91 | <0.01 | Dumas Bay, WA |
| Adenine | C00147 | 9.48E+06 ± 1.24E+06 | 1.66E+06 ± 5.26E+04 | 56.09 | <0.01 | Dumas Bay, WA |
| O.Succinyl.L.Homoserine | C01118 | 1.03E+06 ± 17.17E+04 | 14.11E+04 ± 1.18E+04 | 38.04 | <0.01 | Dumas Bay, WA |
| 3.Dehydroshikimate | C02637 | 1.16E+04 ± 11.51E+02 | 57.56E+02 ± 3.81E+02 | 29.73 | <0.01 | Dumas Bay, WA |
| Disaccharides |  | 4.03E+06 ± 55.30E+04 | 1.52E+06 ± 7.17E+04 | 28.44 | <0.01 | Dumas Bay, WA |
| 4.Acetamidobutanoate | C02946 | 11.89E+04 ± 80.55E+02 | 7.25E+04 ± 48.58E+02 | 27.49 | <0.01 | Dumas Bay, WA |
| Uracil | C00106 | 25.08E+04 ± 4.78E+04 | 3.66E+04 ± 70.89E+02 | 27.43 | <0.01 | Dumas Bay, WA |
| Guanosine | C00387 | 5.57E+04 ± 1.11E+04 | 1.01E+04 ± 8.27E+02 | 24.03 | <0.01 | Dumas Bay, WA |
| 4.Hydroxy.L.Proline | C01157 | 12.54E+04 ± 1.18E+04 | 7.58E+04 ± 30.81E+02 | 22.13 | 0.01 | Dumas Bay, WA |
| Glutaric.Acid | C00489 | 10.32E+04 ± 52.65E+02 | 7.62E+04 ± 37.02E+02 | 18.86 | 0.01 | Dumas Bay, WA |
| Succinate.Semialdehyde | C00232 | 62.22E+02 ± 5.92E+02 | 19.83E+02 ± 6.06E+02 | 25.06 | 0.01 | Dumas Bay, WA |
| Glyceric.Acid (Glycerate) | C00258 | 19.75E+04 ± 1.13E+04 | 15.54E+04 ± 34.57E+02 | 16.74 | 0.01 | Dumas Bay, WA |
| Phloroglucinol | C02183 | 5.25E+06 ± 45.37E+04 | 3.06E+06 ± 32.92E+04 | 16.26 | 0.01 | Dumas Bay, WA |
| D.Arabinose | C00216 | 69.28E+04 ± 3.15E+04 | 26.05E+04 ± 10.75E+04 | 11.08 | 0.02 | Dumas Bay, WA |
| Hypoxanthine | C00262 | 6.68E+04 ± 1.33E+04 | 3.09E+04 ± 29.52E+02 | 9.44 | 0.03 | Dumas Bay, WA |
| Cytosine | C00380 | 60.80E+04 ± 14.70E+04 | 23.99E+04 ± 2.93E+04 | 8.30 | 0.03 | Dumas Bay, WA |
| Quinoline | C06413 | 4.99E+04 ± 16.23E+02 | 3.03E+04 ± 61.21E+02 | 7.10 | 0.04 | Dumas Bay, WA |
| Alpha.Aminoadipate | C00956 | 13.83E+04 ± 2.92E+04 | 6.99E+04 ± 1.05E+04 | 6.24 | 0.05 | Dumas Bay, WA |
| 1.Methyladenine | C02216 | 7.93E+04 ± 52.28E+02 | 4.84E+04 ± 1.00E+04 | 5.97 | 0.06 | Dumas Bay, WA |
| 5.Methylcytosine.Hydrocloride | C02376 | 4.92E+04 ± 43.70E+02 | 2.79E+04 ± 67.57E+02 | 5.89 | 0.06 | Dumas Bay, WA |
| Aspartate | C00049 | 1.43E+06 ± 9.70E+04 | 1.14E+06 ± 7.57E+04 | 5.82 | 0.06 | Dumas Bay, WA |
| Urocanate | C00785 | 14.66E+04 ± 6.40E+04 | 2.94E+04 ± 38.16E+02 | 4.75 | 0.08 | Dumas Bay, WA |
| L.Serine | C00716 | 24.18E+04 ± 7.67E+04 | 10.13E+04 ± 2.15E+04 | 4.15 | 0.10 | Dumas Bay, WA |
| Histamine | C00388 | 6.39E+04 ± 95.39E+02 | 4.01E+04 ± 74.54E+02 | 4.01 | 0.10 | Dumas Bay, WA |
| Sucrose | C00089 | 1.03E+08 ± 9.44E+06 | 85.07E+06 ± 4.28E+06 | 3.70 | 0.11 | Dumas Bay, WA |
| Pyruvate | C00022 | 7.38E+04 ± 69.77E+02 | 5.78E+04 ± 51.92E+02 | 3.55 | 0.12 | Dumas Bay, WA |
| Hexoses.Phosphate |  | 11.35E+04 ± 2.73E+04 | 5.82E+04 ± 1.60E+04 | 3.47 | 0.12 | Dumas Bay, WA |
| Glyceraldehyde | C02154 | 44.02E+04 ± 4.72E+04 | 32.40E+04 ± 4.47E+04 | 3.11 | 0.14 | Dumas Bay, WA |
| Guanine | C00242 | 92.49E+04 ± 26.23E+04 | 49.42E+04 ± 9.82E+04 | 3.01 | 0.14 | Dumas Bay, WA |
| Nalpha.Acetyl.L.Lysine | C12989 | 7.55E+04 ± 95.55E+02 | 6.16E+04 ± 18.15E+02 | 2.81 | 0.15 | Dumas Bay, WA |
| Thymine | C00178 | 8.34E+04 ± 1.38E+04 | 5.86E+04 ± 92.14E+02 | 2.44 | 0.18 | Dumas Bay, WA |
| Rs.Mevalonic.Acid | C00418 | 46.72E+02 ± 12.80E+02 | 29.90E+02 ± 2.90E+02 | 2.24 | 0.19 | Dumas Bay, WA |
| Pyridoxamine | C00534 | 5.83E+04 ± 1.37E+04 | 4.14E+04 ± 44.95E+02 | 1.77 | 0.24 | Dumas Bay, WA |
| Creatine | C00300 | 7.15E+04 ± 3.75E+04 | 2.05E+04 ± 2.78E+02 | 1.85 | 0.25 | Dumas Bay, WA |
| Naringenin | C00509 | 22.46E+02 ± 16.78E+02 | 4.11E+02 ± 1.21E+02 | 1.69 | 0.25 | Dumas Bay, WA |
| 2.Hydroxypyridine | C02502 | 1.27E+06 ± 19.79E+04 | 93.81E+04 ± 16.30E+04 | 1.69 | 0.25 | Dumas Bay, WA |
| L.Alanine | C00041 | 76.48E+04 ± 14.37E+04 | 54.61E+04 ± 11.31E+04 | 1.48 | 0.28 | Dumas Bay, WA |
| Eriodictyol | C05631 | 26.31E+02 ± 20.88E+02 | 5.54E+02 ± 1.24E+02 | 1.40 | 0.29 | Dumas Bay, WA |
| N.Acetyl.L.Alanine | C01073 | 3.65E+04 ± 36.02E+02 | 3.21E+04 ± 20.54E+02 | 1.31 | 0.30 | Dumas Bay, WA |
| Palmitic.Acid | C00249 | 3.25E+06 ± 99.43E+04 | 2.37E+06 ± 14.78E+04 | 1.07 | 0.35 | Dumas Bay, WA |
| Nicotinamide | C00153 | 1.09E+06 ± 15.29E+04 | 91.81E+04 ± 8.78E+04 | 1.07 | 0.35 | Dumas Bay, WA |
| Amino-Sugars |  | 6.37E+04 ± 2.19E+04 | 4.28E+04 ± 86.20E+02 | 1.00 | 0.36 | Dumas Bay, WA |
| Galactitol | C01697 | 60.72E+02 ± 24.15E+02 | 42.59E+02 ± 10.01E+02 | 0.60 | 0.47 | Dumas Bay, WA |
| D.Pantothenic.Acid | C00864 | 6.96E+04 ± 1.51E+04 | 5.53E+04 ± 1.32E+04 | 0.51 | 0.51 | Dumas Bay, WA |
| Turanose | C19636 | 1.41E+06 ± 30.46E+04 | 1.23E+06 ± 8.04E+04 | 0.45 | 0.53 | Dumas Bay, WA |
| 1.2.Phenylenediamine | C14402 | 5.86E+04 ± 1.49E+04 | 4.49E+04 ± 1.39E+04 | 0.45 | 0.53 | Dumas Bay, WA |
| Acetoacetate | C00164 | 5.17E+04 ± 15.66E+02 | 4.98E+04 ± 21.79E+02 | 0.43 | 0.54 | Dumas Bay, WA |
| 4.Hydroxy.L.Phenylglycine.Pyridoxal | CA1445 | 12.21E+04 ± 47.18E+02 | 10.51E+04 ± 2.23E+04 | 0.40 | 0.55 | Dumas Bay, WA |
| N.Acetylglycine | CA1212 | 9.12E+04 ± 1.71E+04 | 7.94E+04 ± 1.12E+04 | 0.37 | 0.57 | Dumas Bay, WA |
| Nicotinate.Picolinic.Acid | C00253 | 4.96E+04 ± 52.35E+02 | 4.43E+04 ± 66.49E+02 | 0.35 | 0.58 | Dumas Bay, WA |
| Tyramine | C00483 | 5.21E+04 ± 1.42E+04 | 4.38E+04 ± 66.93E+02 | 0.34 | 0.58 | Dumas Bay, WA |
| L.Threonine | C00188 | 24.26E+04 ± 5.02E+04 | 21.51E+04 ± 3.01E+04 | 0.25 | 0.64 | Dumas Bay, WA |
| Salsolinol | C09642 | 4.47E+04 ± 1.12E+04 | 3.92E+04 ± 53.76E+02 | 0.23 | 0.65 | Dumas Bay, WA |
| D.Glucuronolactone | C00191 | 12.34E+04 ± 42.83E+02 | 11.61E+04 ± 1.27E+04 | 0.23 | 0.65 | Dumas Bay, WA |
| Shikimate | C00493 | 28.77E+04 ± 2.28E+04 | 26.43E+04 ± 4.10E+04 | 0.20 | 0.67 | Dumas Bay, WA |
| Maleamate | C01596 | 8.13E+04 ± 2.14E+04 | 7.40E+04 ± 73.35E+02 | 0.13 | 0.73 | Dumas Bay, WA |
| 2.Aminophenol | C01987 | 74.03E+04 ± 12.21E+04 | 69.14E+04 ± 11.78E+04 | 0.08 | 0.79 | Dumas Bay, WA |
| Linoleic.Acid | C01595 | 29.20E+04 ± 21.40E+04 | 25.24E+04 ± 3.91E+04 | 0.08 | 0.80 | Dumas Bay, WA |
| Azelaic.Acid | C08261 | 64.63E+02 ± 18.57E+02 | 56.31E+02 ± 26.40E+02 | 0.06 | 0.82 | Dumas Bay, WA |
| 6.Hydroxynicotinate | C01020 | 5.34E+04 ± 75.80E+02 | 5.09E+04 ± 91.03E+02 | 0.04 | 0.85 | Dumas Bay, WA |
| 3.Methoxytyramine | C05587 | 4.69E+04 ± 67.02E+02 | 4.57E+04 ± 49.92E+02 | 0.02 | 0.89 | Dumas Bay, WA |
| Citrate | C00158 | 3.54E+06 ± 66.92E+04 | 3.42E+06 ± 44.11E+04 | 0.02 | 0.89 | Dumas Bay, WA |
| Pyruvic.Aldehyde | C00546 | 10.21E+04 ± 1.06E+04 | 10.17E+04 ± 2.96E+04 | 0.00 | 0.99 | Dumas Bay, WA |
|  |  |  |  |  |  |  |
| Glycerol.3.Phosphate | C00093 | 76.28E+04 ± 13.98E+04 | 1.74E+06 ± 8.31E+04 | 41.28 | <0.01 | South Bay, VA |
| Rosmarinic.Acid | C01850 | 4.21E+04 ± 2.40E+04 | 3.01E+06 ± 39.52E+04 | 40.24 | <0.01 | South Bay, VA |
| Caffeic.Acid | C01197 | 65.42E+04 ± 4.94E+04 | 1.46E+06 ± 10.44E+04 | 38.67 | <0.01 | South Bay, VA |
| Resorcinol.Monoacetate | C12064 | 74.55E+02 ± 23.71E+02 | 4.24E+04 ± 57.48E+02 | 24.27 | <0.01 | South Bay, VA |
| Dehydroascorbate | C05422 | 72.58E+04 ± 2.23E+04 | 1.50E+06 ± 13.47E+04 | 23.41 | <0.01 | South Bay, VA |
| 4.Hydroxybenzaldehyde | C00633 | 11.03E+02 ± 3.06E+02 | 9.77E+04 ± 33.48E+02 | 591.92 | 0.00 | South Bay, VA |
| Adenosine | C00212 | 7.69E+04 ± 1.04E+04 | 8.28E+06 ± 43.48E+04 | 253.90 | 0.00 | South Bay, VA |
| Myo.Inositol | C00137 | 32.52E+06 ± 3.90E+06 | 56.09E+06 ± 3.34E+06 | 21.15 | 0.01 | South Bay, VA |
| N.Acetyl.Dl.Glutamic.Acid | C00624 | 1.40E+06 ± 37.90E+04 | 5.97E+06 ± 84.76E+04 | 18.90 | 0.01 | South Bay, VA |
| 1.Aminocyclopropane.1.Carboxylate | C01234 | 1.87E+06 ± 51.08E+04 | 6.70E+06 ± 87.71E+04 | 18.47 | 0.01 | South Bay, VA |
| Nepsilon.Nepsilon.Nepsilon.Trimethyllysine | C03793 | 34.27E+02 ± 14.85E+02 | 76.21E+02 ± 3.70E+02 | 15.49 | 0.02 | South Bay, VA |
| Formononetin | C00858 | 6.55E+02 ± 2.59E+02 | 40.87E+02 ± 8.71E+02 | 10.63 | 0.02 | South Bay, VA |
| L.Proline | C16435 | 46.84E+06 ± 8.59E+06 | 74.92E+06 ± 4.57E+06 | 9.74 | 0.03 | South Bay, VA |
| Adenosine.5.Monophosphate | C00020 | 5.87E+04 ± 2.20E+04 | 32.22E+04 ± 6.97E+04 | 9.73 | 0.03 | South Bay, VA |
| L.Asparagine | C16438 | 14.46E+04 ± 7.41E+04 | 50.54E+04 ± 8.46E+04 | 9.40 | 0.03 | South Bay, VA |
| L.Isoleucine | C16434 | 1.41E+06 ± 18.19E+04 | 2.77E+06 ± 35.80E+04 | 9.21 | 0.03 | South Bay, VA |
| L.Tyrosine | C01536 | 28.04E+04 ± 43.56E+02 | 47.13E+04 ± 5.62E+04 | 8.22 | 0.04 | South Bay, VA |
| L.DOPA | C00355 | 39.15E+04 ± 10.68E+04 | 3.69E+06 ± 72.90E+04 | 9.05 | 0.04 | South Bay, VA |
| 3.Hydroxykynurenine | C02794 | 7.02E+04 ± 1.69E+04 | 19.42E+04 ± 3.83E+04 | 6.82 | 0.05 | South Bay, VA |
| a.Ketoglutaric.Acid | C00026 | 6.06E+04 ± 27.71E+02 | 11.03E+04 ± 1.65E+04 | 6.41 | 0.05 | South Bay, VA |
| L.Sorbose | C00247 | 8.06E+06 ± 88.34E+04 | 18.85E+06 ± 3.73E+06 | 5.83 | 0.06 | South Bay, VA |
| Salicylate | C00805 | 25.02E+02 ± 7.96E+02 | 6.99E+04 ± 2.37E+04 | 5.78 | 0.06 | South Bay, VA |
| Fructose | C00095 | 13.02E+06 ± 1.54E+06 | 32.97E+06 ± 7.02E+06 | 5.64 | 0.06 | South Bay, VA |
| L.Glutamine | C00303 | 18.46E+06 ± 9.55E+06 | 54.51E+06 ± 11.13E+06 | 5.47 | 0.07 | South Bay, VA |
| Monoshaccharides Hexoses |  | 61.80E+04 ± 4.49E+04 | 98.34E+04 ± 13.27E+04 | 5.12 | 0.07 | South Bay, VA |
| 5.Oxo.L.Proline | C01879 | 11.23E+06 ± 4.36E+06 | 27.71E+06 ± 5.33E+06 | 5.11 | 0.07 | South Bay, VA |
| Fisetin | C10041 | 80.10E+06 ± 5.36E+06 | 98.82E+06 ± 5.89E+06 | 5.10 | 0.07 | South Bay, VA |
| Glutamic.Acid | C00025 | 8.71E+06 ± 1.20E+06 | 15.68E+06 ± 2.57E+06 | 4.73 | 0.08 | South Bay, VA |
| Sugars.Alcohol.Pentoses |  | 83.83E+02 ± 18.06E+02 | 1.39E+04 ± 18.22E+02 | 4.42 | 0.09 | South Bay, VA |
| Gallic.Acid | C01424 | 23.49E+02 ± 7.62E+02 | 52.66E+02 ± 10.73E+02 | 4.21 | 0.10 | South Bay, VA |
| Succinate | C00042 | 13.28E+04 ± 1.81E+04 | 20.83E+04 ± 2.86E+04 | 4.15 | 0.10 | South Bay, VA |
| 5.Methylthioadenosine | C00170 | 2.62E+04 ± 1.08E+04 | 8.08E+04 ± 2.14E+04 | 4.11 | 0.10 | South Bay, VA |
| S.1.Phenylethanol | C07112 | 48.96E+04 ± 3.52E+04 | 80.18E+04 ± 13.17E+04 | 3.88 | 0.11 | South Bay, VA |
| L.Pipecolic.Acid | C00408 | 95.68E+04 ± 6.52E+04 | 1.38E+06 ± 18.17E+04 | 3.71 | 0.11 | South Bay, VA |
| Luteolin | C01514 | 3.72E+06 ± 1.47E+06 | 7.19E+06 ± 1.29E+06 | 3.12 | 0.14 | South Bay, VA |
| D.Malic.Acid | C00497 | 2.43E+06 ± 85.22E+04 | 5.12E+06 ± 1.23E+06 | 2.73 | 0.16 | South Bay, VA |
| D.Mannose | C00159 | 53.57E+04 ± 3.74E+04 | 75.02E+04 ± 10.90E+04 | 2.61 | 0.17 | South Bay, VA |
| Pyridoxine | C00314 | 11.70E+04 ± 2.41E+04 | 15.62E+04 ± 1.17E+04 | 2.56 | 0.17 | South Bay, VA |
| S.Malate | C00711 | 3.31E+06 ± 1.07E+06 | 6.95E+06 ± 1.86E+06 | 2.34 | 0.19 | South Bay, VA |
| Trigonelline | C01004 | 7.09E+06 ± 2.27E+06 | 10.31E+06 ± 99.40E+04 | 2.08 | 0.21 | South Bay, VA |
| Mandelic.Acid | C01984 | 1.82E+04 ± 41.11E+02 | 3.52E+04 ± 1.01E+04 | 1.89 | 0.23 | South Bay, VA |
| D.Lyxosylamine |  | 1.33E+06 ± 12.64E+04 | 1.70E+06 ± 22.23E+04 | 1.67 | 0.25 | South Bay, VA |
| 3.Aminoisobutanoate | C05145 | 2.32E+04 ± 27.27E+02 | 3.49E+04 ± 74.17E+02 | 1.67 | 0.25 | South Bay, VA |
| N.Acetyl.D.Tryptophan | C03137 | 1.12E+04 ± 39.55E+02 | 22.26E+04 ± 14.67E+04 | 1.48 | 0.28 | South Bay, VA |
| 3.Amino.5.Hydroxybenzoic.Acid | C12107 | 3.78E+04 ± 60.36E+02 | 5.52E+04 ± 1.20E+04 | 1.33 | 0.30 | South Bay, VA |
| Xylitol | C00379 | 7.99E+04 ± 1.90E+04 | 10.07E+04 ± 1.03E+04 | 1.08 | 0.35 | South Bay, VA |
| L.Valine | C00183 | 4.33E+06 ± 2.19E+06 | 7.29E+06 ± 1.92E+06 | 1.03 | 0.36 | South Bay, VA |
| Leucine | C16439 | 44.94E+04 ± 8.92E+04 | 53.67E+04 ± 4.00E+04 | 0.98 | 0.37 | South Bay, VA |
| Uridine | C00299 | 5.69E+04 ± 99.28E+02 | 7.03E+04 ± 93.64E+02 | 0.93 | 0.38 | South Bay, VA |
| Galactonic.Acid | C00880 | 59.85E+04 ± 26.28E+04 | 79.71E+04 ± 4.46E+04 | 0.77 | 0.42 | South Bay, VA |
| 3.Amino.4.Hydroxybenzoic.Acid | C12115 | 4.66E+04 ± 2.06E+04 | 6.38E+04 ± 87.54E+02 | 0.73 | 0.43 | South Bay, VA |
| L.Phenylalanine | C02057 | 73.54E+04 ± 7.22E+04 | 99.61E+04 ± 26.35E+04 | 0.67 | 0.45 | South Bay, VA |
| 6.Phosphogluconic.Acid | C00345 | 5.00E+04 ± 58.99E+02 | 6.33E+04 ± 1.60E+04 | 0.46 | 0.53 | South Bay, VA |
| 2.6.Dihydroxypyridine | C03056 | 6.49E+04 ± 1.26E+04 | 8.19E+04 ± 2.24E+04 | 0.35 | 0.58 | South Bay, VA |
| Fumarate | C00122 | 18.25E+04 ± 3.90E+04 | 20.79E+04 ± 2.70E+04 | 0.31 | 0.60 | South Bay, VA |
| Monosaccharides Pentoses |  | 9.74E+04 ± 90.09E+02 | 10.81E+04 ± 1.81E+04 | 0.22 | 0.66 | South Bay, VA |
| Deoxy-Hexoses |  | 70.73E+02 ± 10.30E+02 | 82.95E+02 ± 23.56E+02 | 0.18 | 0.69 | South Bay, VA |
| 4.Aminobutanoate (GABA) | C00334 | 32.04E+04 ± 49.08E+02 | 39.00E+04 ± 14.84E+04 | 0.16 | 0.71 | South Bay, VA |
| 4.Guanidinobutanoate | C01035 | 5.41E+04 ± 2.22E+04 | 6.27E+04 ± 1.31E+04 | 0.13 | 0.73 | South Bay, VA |
| Arabitol | C01904 | 42.47E+04 ± 3.60E+04 | 45.17E+04 ± 6.94E+04 | 0.10 | 0.77 | South Bay, VA |
| Sugars.Alcohol.Hexoses |  | 75.83E+02 ± 17.26E+02 | 80.87E+02 ± 8.58E+02 | 0.08 | 0.79 | South Bay, VA |
| D.Trehalose | C01083 | 1.13E+06 ± 31.01E+04 | 1.18E+06 ± 8.40E+04 | 0.03 | 0.88 | South Bay, VA |
| Creatinine | C00791 | 3.48E+04 ± 67.41E+02 | 3.62E+04 ± 1.14E+04 | 0.01 | 0.93 | South Bay, VA |
| 3.2.Hydroxyphenyl.Propanoate | C01198 | 80.47E+02 ± 18.64E+02 | 82.21E+02 ± 14.71E+02 | 0.01 | 0.94 | South Bay, VA |
| D.Gulonic.Acid.Gama.Lactone | C01040 | 2.92E+04 ± 42.98E+02 | 2.95E+04 ± 69.92E+02 | 0.00 | 0.97 | South Bay, VA |
| L.Arginine | C00062 | 1.07E+04 ± 43.58E+02 | 1.07E+04 ± 31.86E+02 | 0.00 | 0.99 | South Bay, VA |

**Table S8**. Summary PERMANOVA results for effects of [CO_2_] on leaf metabolites for South Bay VA and Dumas Bay WA separately.

|  | **Source** | ***df*** | **Sum of Squares** | **Mean Square** | ***F*** | ***p*** |
| --- | --- | --- | --- | --- | --- | --- |
| All CO_2_ SBV | [CO_2_] | 1 | 1.59 x 10^17^ | 1.59 x 10^17^ | 5.53 | <0.001* |
| All CO2 DB | [CO_2_] | 1 | 7.77 x 10^16^ | 7.77 x 10^16^ | 4.03 | 0.1 |

**Table S9.** ANOVA CO_2_ treatment comparison of identified leaf metabolites relative abundance (i.e. MS peak area) and standard error of South Bay eelgrass. Highest CO_2_ (2121 µmol CO_2_ kgSW^-1^), Ambient CO_2_ (55 µmol CO_2_ kgSW^-1^)

| **Metabolite** | **KEGG ID** | **Highest CO2**  **Mean ± SE MS Peak Area** | **Ambient CO2**  **Mean ± SE MS Peak Area** | ***F*** | ***p*** | | **Higher concentration** |
| --- | --- | --- | --- | --- | --- | --- | --- |
| L.DOPA | C00355 | 5.13E+06 ± 47.79E+04 | 1.07E+06 ± 5.46E+04 | 51.16 | < 0.01 | | Highest CO_2_ |
| Monosaccharides Pentoses |  | 16.82E+04 ± 52.20E+02 | 12.91E+04 ± 28.38E+02 | 35.02 | < 0.01 | | Highest CO_2_ |
| Linoleic.Acid | C01595 | 26.87E+04 ± 1.92E+04 | 11.49E+04 ± 1.68E+04 | 33.28 | < 0.01 | | Highest CO_2_ |
| Caffeic.Acid | C01197 | 2.27E+06 ± 11.85E+04 | 1.08E+06 ± 19.73E+04 | 30.12 | < 0.01 | | Highest CO_2_ |
| Galactonic.Acid | C00880 | 1.21E+06 ± 6.58E+04 | 73.40E+04 ± 5.07E+04 | 28.53 | < 0.01 | | Highest CO_2_ |
| Rosmarinic.Acid | C01850 | 7.14E+06 ± 94.33E+04 | 1.42E+06 ± 33.84E+04 | 24.66 | < 0.01 | | Highest CO_2_ |
| Myo.Inositol | C00137 | 78.23E+06 ± 6.55E+06 | 42.65E+06 ± 1.17E+06 | 20.75 | 0.01 | | Highest CO_2_ |
| D.Mannose | C00159 | 1.58E+06 ± 19.49E+04 | 83.71E+04 ± 1.93E+04 | 10.42 | 0.02 | | Highest CO_2_ |
| L.Phenylalanine | C02057 | 8.82E+06 ± 1.72E+06 | 2.61E+06 ± 8.03E+04 | 9.30 | 0.03 | | Highest CO_2_ |
| L.Sorbose | C00247 | 46.69E+06 ± 7.63E+06 | 20.50E+06 ± 1.33E+06 | 8.30 | 0.03 | | Highest CO_2_ |
| Glutamic.Acid (Glutamate) | C00025 | 29.48E+06 ± 6.07E+06 | 8.98E+06 ± 1.10E+06 | 8.02 | 0.04 | | Highest CO_2_ |
| Fructose | C00095 | 76.32E+06 ± 11.71E+06 | 36.77E+06 ± 2.67E+06 | 7.94 | 0.04 | | Highest CO_2_ |
| Xylitol | C00379 | 22.79E+04 ± 3.26E+04 | 11.71E+04 ± 1.47E+04 | 7.48 | 0.04 | | Highest CO_2_ |
| D.Arabinose | C00216 | 31.37E+04 ± 5.80E+04 | 12.54E+04 ± 97.67E+02 | 7.42 | 0.04 | | Highest CO_2_ |
| Trigonelline | C01004 | 14.13E+06 ± 1.02E+06 | 7.81E+06 ± 2.43E+06 | 7.17 | 0.04 | | Highest CO_2_ |
| 6.Phosphogluconic.Acid (Gluconate 6P) | C00345 | 72.25E+04 ± 20.81E+04 | 7.51E+04 ± 3.12E+04 | 6.84 | 0.05 | | Highest CO_2_ |
| Luteolin | C01514 | 8.12E+06 ± 1.51E+06 | 3.38E+06 ± 53.98E+04 | 6.60 | 0.05 | | Highest CO_2_ |
| Creatine | C00300 | 4.31E+04 ± 1.31E+04 | 55.22E+02 ± 15.97E+02 | 5.88 | 0.06 | | Highest CO_2_ |
| Nepsilon.Trimethyllysine | C03793 | 4.58E+04 ± 82.91E+02 | 1.84E+04 ± 97.15E+02 | 4.61 | 0.08 | | Highest CO_2_ |
| N.Acetyl.D.Tryptophan | C03137 | 2.86E+04 ± 45.99E+02 | 1.78E+04 ± 9.11E+02 | 3.87 | 0.11 | | Highest CO_2_ |
| Resorcinol.Monoacetate | C12064 | 3.84E+04 ± 29.14E+02 | 2.75E+04 ± 53.68E+02 | 3.73 | 0.11 | | Highest CO_2_ |
| 5.Oxo.L.Proline | C01879 | 24.76E+06 ± 6.37E+06 | 10.12E+06 ± 3.43E+06 | 3.30 | 0.13 | | Highest CO_2_ |
| 1.Aminocyclopropane.1.Carboxylate | C01234 | 8.02E+06 ± 2.74E+06 | 2.21E+06 ± 1.05E+06 | 3.00 | 0.14 | | Highest CO_2_ |
| L.Proline | C16435 | 1.31E+08 ± 33.30E+06 | 62.48E+06 ± 7.49E+06 | 2.98 | 0.14 | | Highest CO_2_ |
| D.Trehalose | C01083 | 2.52E+06 ± 58.08E+04 | 1.37E+06 ± 9.53E+04 | 2.73 | 0.16 | | Highest CO_2_ |
| L.Glutamine | C00303 | 40.99E+06 ± 10.35E+06 | 18.69E+06 ± 8.50E+06 | 2.48 | 0.18 | | Highest CO_2_ |
| Deoxy-Hexoses |  | 1.56E+04 ± 29.60E+02 | 99.06E+02 ± 16.73E+02 | 2.29 | 0.19 | | Highest CO_2_ |
| Creatinine | C00791 | 4.13E+04 ± 81.63E+02 | 2.71E+04 ± 6.14E+02 | 2.17 | 0.20 | | Highest CO_2_ |
| L.Threonine | C00188 | 17.80E+04 ± 1.02E+04 | 15.95E+04 ± 59.01E+02 | 2.00 | 0.22 | | Highest CO_2_ |
| 4.Hydroxybenzaldehyde | C00633 | 10.41E+04 ± 3.44E+04 | 5.41E+04 ± 1.99E+04 | 1.29 | 0.31 | | Highest CO_2_ |
| S.1.Phenylethanol | C07112 | 74.30E+04 ± 20.39E+04 | 48.61E+04 ± 5.36E+04 | 1.10 | 0.34 | | Highest CO_2_ |
| L.Valine | C00183 | 4.71E+06 ± 1.00E+06 | 3.49E+06 ± 29.82E+04 | 1.02 | 0.36 | | Highest CO_2_ |
| Turanose | C19636 | 2.56E+06 ± 75.49E+04 | 1.69E+06 ± 10.71E+04 | 0.95 | 0.37 | | Highest CO_2_ |
| L.Serine | C00716 | 42.49E+04 ± 15.27E+04 | 23.76E+04 ± 8.37E+04 | 0.93 | 0.38 | | Highest CO_2_ |
| Pyruvic.Aldehyde | C00546 | 18.07E+04 ± 3.84E+04 | 13.58E+04 ± 1.61E+04 | 0.90 | 0.39 | | Highest CO_2_ |
| L.Asparagine | C16438 | 23.63E+04 ± 3.78E+04 | 16.61E+04 ± 7.39E+04 | 0.85 | 0.40 | | Highest CO_2_ |
| Arabitol | C01904 | 60.18E+04 ± 9.25E+04 | 52.83E+04 ± 1.93E+04 | 0.44 | 0.54 | | Highest CO_2_ |
| Shikimate | C00493 | 17.18E+04 ± 2.09E+04 | 14.15E+04 ± 5.84E+04 | 0.31 | 0.60 | | Highest CO_2_ |
| Monoshaccharides Hexoses |  | 1.13E+06 ± 25.53E+04 | 96.21E+04 ± 9.44E+04 | 0.27 | 0.62 | | Highest CO_2_ |
| Succinate.Semialdehyde | C00232 | 42.20E+02 ± 3.68E+02 | 39.65E+02 ± 5.07E+02 | 0.18 | 0.69 | | Highest CO_2_ |
| Aspartate | C00049 | 81.66E+04 ± 13.85E+04 | 73.31E+04 ± 17.10E+04 | 0.15 | 0.72 | | Highest CO_2_ |
| Salicylate | C00805 | 2.03E+04 ± 52.65E+02 | 1.75E+04 ± 65.44E+02 | 0.12 | 0.75 | | Highest CO_2_ |
| 3.Aminoisobutanoate | C05145 | 3.06E+04 ± 23.08E+02 | 2.93E+04 ± 78.47E+02 | 0.03 | 0.86 | | Highest CO_2_ |
| Succinate | C00042 | 17.01E+04 ± 43.32E+02 | 16.81E+04 ± 1.70E+04 | 0.02 | 0.90 | | Highest CO_2_ |
| L.Isoleucine | C16434 | 6.76E+06 ± 55.15E+04 | 6.63E+06 ± 1.03E+06 | 0.01 | 0.91 | | Highest CO_2_ |
| 3.2.Hydroxyphenyl.Propanoate | C01198 | 1.04E+04 ± 5.95E+02 | 1.03E+04 ± 8.24E+02 | 0.01 | 0.94 | | Highest CO_2_ |
|  |  |  |  |  |  |  | |
| Cytosine | C00380 | 3.16E+04 ± 44.55E+02 | 30.85E+04 ± 82.01E+02 | 1024.49 | < 0.01 | | Ambient CO_2_ |
| Guanosine | C00387 | 44.35E+02 ± 1.53E+02 | 3.45E+04 ± 5.54E+02 | 4324.15 | < 0.01 | | Ambient CO_2_ |
| Thymine | C00178 | 1.50E+04 ± 20.81E+02 | 8.47E+04 ± 39.04E+02 | 290.69 | < 0.01 | | Ambient CO_2_ |
| D.Glucuronolactone | C00191 | 4.05E+04 ± 29.29E+02 | 10.25E+04 ± 23.42E+02 | 242.38 | < 0.01 | | Ambient CO_2_ |
| 2.Aminophenol | C01987 | 19.60E+04 ± 5.29E+04 | 1.13E+06 ± 21.53E+02 | 224.37 | < 0.01 | | Ambient CO_2_ |
| Urocanate | C00785 | 55.89E+02 ± 14.91E+02 | 4.67E+04 ± 25.17E+02 | 223.84 | < 0.01 | | Ambient CO_2_ |
| Adenosine | C00212 | 31.89E+04 ± 16.43E+04 | 10.61E+06 ± 79.52E+04 | 220.62 | < 0.01 | | Ambient CO_2_ |
| 2.Hydroxypyridine | C02502 | 19.71E+04 ± 3.44E+04 | 1.31E+06 ± 7.92E+04 | 204.94 | < 0.01 | | Ambient CO_2_ |
| Guanine | C00242 | 5.34E+04 ± 38.76E+02 | 55.92E+04 ± 4.55E+04 | 174.26 | < 0.01 | | Ambient CO_2_ |
| 3.Amino.4.Hydroxybenzoic.Acid | C12115 | 1.34E+04 ± 39.38E+02 | 10.72E+04 ± 65.62E+02 | 169.69 | < 0.01 | | Ambient CO_2_ |
| 5.Methylthioadenosine | C00170 | 2.70E+04 ± 6.58E+02 | 22.55E+04 ± 1.10E+04 | 575.99 | < 0.01 | | Ambient CO_2_ |
| Pyridoxine | C00314 | 7.89E+04 ± 1.21E+04 | 24.94E+04 ± 1.04E+04 | 103.07 | < 0.01 | | Ambient CO_2_ |
| D.Pantothenic.Acid | C00864 | 1.43E+04 ± 33.91E+02 | 9.83E+04 ± 89.01E+02 | 98.50 | < 0.01 | | Ambient CO_2_ |
| Eriodictyol | C05631 | 1.54E+02 ± 9.27E+00 | 6.90E+02 ± 27.38E+00 | 513.31 | < 0.01 | | Ambient CO_2_ |
| N.Acetyl.L.Alanine | C01073 | 1.12E+04 ± 14.02E+02 | 3.91E+04 ± 28.20E+02 | 94.20 | < 0.01 | | Ambient CO_2_ |
| Nicotinamide | C00153 | 67.28E+04 ± 3.45E+04 | 1.23E+06 ± 4.90E+04 | 93.67 | < 0.01 | | Ambient CO_2_ |
| 4.Hydroxy.L.Phenylglycine.Pyridoxal | CA1445 | 7.51E+04 ± 1.65E+04 | 25.55E+04 ± 59.20E+02 | 80.58 | < 0.01 | | Ambient CO_2_ |
| Hypoxanthine | C00262 | 66.08E+02 ± 19.69E+02 | 3.32E+04 ± 22.32E+02 | 79.25 | < 0.01 | | Ambient CO_2_ |
| Tyramine | C00483 | 4.37E+04 ± 41.78E+02 | 10.22E+04 ± 56.21E+02 | 73.64 | < 0.01 | | Ambient CO_2_ |
| Histamine | C00388 | 1.69E+04 ± 44.53E+02 | 9.10E+04 ± 15.14E+02 | 120.80 | < 0.01 | | Ambient CO_2_ |
| Salsolinol | C09642 | 2.12E+04 ± 35.26E+02 | 6.81E+04 ± 50.90E+02 | 61.97 | < 0.01 | | Ambient CO_2_ |
| Maleamate | C01596 | 2.58E+04 ± 12.88E+02 | 4.86E+04 ± 30.21E+02 | 59.59 | < 0.01 | | Ambient CO_2_ |
| Glyceraldehyde | C02154 | 25.41E+04 ± 1.13E+04 | 49.55E+04 ± 3.70E+04 | 51.31 | < 0.01 | | Ambient CO_2_ |
| Glutaric.Acid | C00489 | 6.24E+04 ± 55.75E+02 | 12.46E+04 ± 76.44E+02 | 45.90 | < 0.01 | | Ambient CO_2_ |
| 1.2.Phenylenediamine | C14402 | 2.57E+04 ± 84.42E+02 | 10.08E+04 ± 83.99E+02 | 37.78 | < 0.01 | | Ambient CO_2_ |
| Naringenin | C00509 | 1.60E+02 ± 71.44E+00 | 6.76E+02 ± 13.09E+00 | 36.53 | < 0.01 | | Ambient CO_2_ |
| 3.Amino.5.Hydroxybenzoic.Acid | C12107 | 2.12E+04 ± 22.92E+02 | 4.87E+04 ± 45.66E+02 | 34.24 | < 0.01 | | Ambient CO_2_ |
| Sugars.Alcohol.Hexoses |  | 22.31E+02 ± 3.69E+02 | 77.14E+02 ± 9.93E+02 | 34.13 | < 0.01 | | Ambient CO_2_ |
| Adenosine.5.Monophosphate | C00020 | 6.31E+04 ± 1.86E+04 | 23.51E+04 ± 2.44E+04 | 32.89 | < 0.01 | | Ambient CO_2_ |
| 6.Hydroxynicotinate | C01020 | 94.13E+02 ± 23.22E+02 | 5.99E+04 ± 1.02E+04 | 31.92 | < 0.01 | | Ambient CO_2_ |
| 1.Methyladenine | C02216 | 2.68E+04 ± 55.05E+02 | 6.86E+04 ± 48.00E+02 | 29.85 | < 0.01 | | Ambient CO_2_ |
| D.Gulonic.Acid.Gama.Lactone | C01040 | 2.17E+04 ± 14.34E+02 | 3.13E+04 ± 9.96E+02 | 25.68 | < 0.01 | | Ambient CO_2_ |
| Sugars.Alcohol.Pentoses |  | 33.23E+02 ± 5.33E+02 | 1.14E+04 ± 17.49E+02 | 25.60 | < 0.01 | | Ambient CO_2_ |
| Amino-Sugars |  | 1.77E+04 ± 60.08E+02 | 8.69E+04 ± 1.43E+04 | 24.61 | < 0.01 | | Ambient CO_2_ |
| Citrate | C00158 | 1.88E+06 ± 25.22E+04 | 3.93E+06 ± 35.57E+04 | 23.57 | < 0.01 | | Ambient CO_2_ |
| Leucine | C16439 | 48.82E+04 ± 5.98E+04 | 89.47E+04 ± 6.07E+04 | 21.76 | 0.01 | | Ambient CO_2_ |
| Glycerol.3.Phosphate | C00093 | 63.35E+04 ± 18.22E+04 | 1.53E+06 ± 3.40E+04 | 16.93 | 0.01 | | Ambient CO_2_ |
| 5.Methylcytosine.Hydrocloride | C02376 | 47.54E+02 ± 10.19E+02 | 3.37E+04 ± 92.60E+02 | 13.66 | 0.01 | | Ambient CO_2_ |
| 4.Acetamidobutanoate (GABA) | C02946 | 5.77E+04 ± 1.41E+04 | 12.22E+04 ± 92.67E+02 | 12.23 | 0.02 | | Ambient CO_2_ |
| a.Ketoglutaric.Acid | C00026 | 5.85E+04 ± 53.78E+02 | 11.31E+04 ± 1.84E+04 | 10.79 | 0.02 | | Ambient CO_2_ |
| Pyruvate | C00022 | 4.19E+04 ± 61.45E+02 | 6.68E+04 ± 28.93E+02 | 10.59 | 0.02 | | Ambient CO_2_ |
| Mandelic.Acid | C01984 | 78.07E+02 ± 11.74E+02 | 2.11E+04 ± 46.46E+02 | 10.30 | 0.02 | | Ambient CO_2_ |
| 4.Guanidinobutanoate | C01035 | 1.27E+04 ± 78.72E+02 | 4.33E+04 ± 24.89E+02 | 10.27 | 0.02 | | Ambient CO_2_ |
| Pyridoxamine | C00534 | 2.61E+04 ± 1.10E+04 | 7.83E+04 ± 1.20E+04 | 10.17 | 0.02 | | Ambient CO_2_ |
| N.Acetylglycine | CA1212 | 4.90E+04 ± 1.34E+04 | 9.87E+04 ± 51.12E+02 | 9.12 | 0.03 | | Ambient CO_2_ |
| Nicotinate.Picolinic.Acid | C00253 | 2.28E+04 ± 15.19E+02 | 4.77E+04 ± 98.05E+02 | 8.82 | 0.03 | | Ambient CO_2_ |
| Alpha.Aminoadipate | C00956 | 4.69E+04 ± 1.16E+04 | 15.62E+04 ± 4.35E+04 | 7.90 | 0.04 | | Ambient CO_2_ |
| 4.Aminobutanoate (GABA) | C00334 | 80.28E+04 ± 24.67E+04 | 2.48E+06 ± 63.52E+04 | 7.61 | 0.04 | | Ambient CO_2_ |
| Acetoacetate | C00164 | 3.74E+04 ± 35.81E+02 | 5.55E+04 ± 64.98E+02 | 6.92 | 0.05 | | Ambient CO_2_ |
| Galactitol | C01697 | 49.38E+02 ± 5.95E+02 | 1.52E+04 ± 49.40E+02 | 5.95 | 0.06 | | Ambient CO_2_ |
| O.Succinyl.L.Homoserine | C01118 | 18.84E+04 ± 1.98E+04 | 49.28E+04 ± 15.12E+04 | 5.59 | 0.06 | | Ambient CO_2_ |
| Hexoses.Phosphate |  | 2.15E+04 ± 12.30E+02 | 5.16E+04 ± 1.54E+04 | 5.42 | 0.07 | | Ambient CO_2_ |
| Phloroglucinol | C02183 | 3.32E+06 ± 44.64E+04 | 4.64E+06 ± 25.71E+04 | 5.38 | 0.07 | | Ambient CO_2_ |
| 3.Dehydroshikimate | C02637 | 43.51E+02 ± 8.57E+02 | 72.92E+02 ± 10.74E+02 | 4.71 | 0.08 | | Ambient CO_2_ |
| Fumarate | C00122 | 18.37E+04 ± 2.16E+04 | 26.64E+04 ± 3.39E+04 | 4.69 | 0.08 | | Ambient CO_2_ |
| Uracil | C00106 | 8.64E+04 ± 2.49E+04 | 15.17E+04 ± 87.87E+02 | 4.62 | 0.08 | | Ambient CO_2_ |
| D.Lyxosylamine |  | 1.58E+06 ± 22.53E+04 | 2.26E+06 ± 20.14E+04 | 4.56 | 0.09 | | Ambient CO_2_ |
| Rs.Mevalonic.Acid | C00418 | 21.90E+02 ± 2.27E+02 | 1.01E+04 ± 45.29E+02 | 4.34 | 0.09 | | Ambient CO_2_ |
| 2.6.Dihydroxypyridine | C03056 | 3.85E+04 ± 1.26E+04 | 7.14E+04 ± 61.08E+02 | 4.33 | 0.09 | | Ambient CO_2_ |
| Disaccharides |  | 1.49E+06 ± 28.29E+04 | 2.21E+06 ± 12.14E+04 | 4.30 | 0.09 | | Ambient CO_2_ |
| L.Tyrosine | C01536 | 42.20E+04 ± 4.82E+04 | 90.44E+04 ± 30.01E+04 | 3.51 | 0.12 | | Ambient CO_2_ |
| 4.Hydroxy.L.Proline | C01157 | 2.26E+04 ± 50.59E+02 | 3.48E+04 ± 66.18E+02 | 2.24 | 0.19 | | Ambient CO_2_ |
| L.Alanine | C00041 | 98.89E+04 ± 5.06E+04 | 1.09E+06 ± 4.54E+04 | 2.06 | 0.21 | | Ambient CO_2_ |
| Quinoline | C06413 | 6.69E+04 ± 1.94E+04 | 10.29E+04 ± 1.59E+04 | 1.84 | 0.23 | | Ambient CO_2_ |
| D.Malic.Acid | C00497 | 1.88E+06 ± 13.19E+04 | 2.93E+06 ± 97.64E+04 | 1.60 | 0.26 | | Ambient CO_2_ |
| Uridine | C00299 | 54.42E+02 ± 16.16E+02 | 1.15E+04 ± 54.23E+02 | 1.51 | 0.27 | | Ambient CO_2_ |
| Fisetin | C10041 | 84.56E+06 ± 6.70E+06 | 93.98E+06 ± 4.67E+06 | 1.14 | 0.34 | | Ambient CO_2_ |
| Glyceric.Acid | C00258 | 14.12E+04 ± 3.54E+04 | 18.07E+04 ± 2.06E+04 | 0.76 | 0.42 | | Ambient CO_2_ |
| Dehydroascorbate | C05422 | 1.33E+06 ± 19.49E+04 | 1.49E+06 ± 11.31E+04 | 0.42 | 0.54 | | Ambient CO_2_ |
| L.Pipecolic.Acid | C00408 | 93.99E+04 ± 13.27E+04 | 1.12E+06 ± 26.31E+04 | 0.42 | 0.54 | | Ambient CO_2_ |
| Adenine | C00147 | 1.59E+06 ± 47.21E+04 | 1.92E+06 ± 26.07E+04 | 0.30 | 0.60 | | Ambient CO_2_ |
| 3.Hydroxykynurenine | C02794 | 6.24E+04 ± 2.10E+04 | 7.81E+04 ± 2.37E+04 | 0.24 | 0.64 | | Ambient CO_2_ |
| 3.Methoxytyramine | C05587 | 4.04E+04 ± 63.19E+02 | 4.56E+04 ± 1.24E+04 | 0.17 | 0.70 | | Ambient CO_2_ |
| Azelaic.Acid | C08261 | 49.95E+02 ± 22.87E+02 | 57.68E+02 ± 3.73E+02 | 0.08 | 0.79 | | Ambient CO_2_ |
| N.Acetyl.Dl.Glutamic.Acid | C00624 | 2.53E+06 ± 51.14E+04 | 2.82E+06 ± 1.05E+06 | 0.08 | 0.80 | | Ambient CO_2_ |
| Nalpha.Acetyl.L.Lysine | C12989 | 4.15E+04 ± 1.32E+04 | 4.57E+04 ± 67.02E+02 | 0.06 | 0.81 | | Ambient CO_2_ |
| Gallic.Acid | C01424 | 46.84E+02 ± 10.44E+02 | 49.65E+02 ± 6.04E+02 | 0.04 | 0.84 | | Ambient CO_2_ |
| L.Arginine | C00062 | 2.68E+04 ± 16.85E+02 | 2.82E+04 ± 94.34E+02 | 0.03 | 0.87 | | Ambient CO_2_ |
| Sucrose | C00089 | 1.16E+08 ± 15.23E+06 | 1.18E+08 ± 9.18E+06 | 0.00 | 0.95 | | Ambient CO_2_ |
| Formononetin | C00858 | 19.92E+02 ± 2.86E+02 | 20.15E+02 ± 7.69E+02 | 0.00 | 0.98 | | Ambient CO_2_ |
| Palmitic.Acid | C00249 | 2.34E+06 ± 33.78E+04 | 2.34E+06 ± 5.60E+04 | 0.00 | 1.00 | | Ambient CO_2_ |
| S.Malate | C00711 | 3.59E+06 ± 60.23E+04 | 3.59E+06 ± 98.41E+04 | 0.00 | 1.00 | | Ambient CO_2_ |

**Table S10**. ANOVA CO_2_ treatment comparison of identified leaf metabolites relative abundance (i.e. MS peak area) and standard error of Dumas Bay eelgrass. High CO_2_ (823 µmol CO_2_ kgSW^-1^), Low CO_2_ (107 µmol CO_2_ kgSW^-1^)

| **Metabolite** | **KEGG ID** | **High CO_2_**  **Mean ± SE MS Peak Area** | **Low CO_2_**  **Mean ± SE MS Peak Area** | ***F*** | ***p*** | **Higher concentration** |
| --- | --- | --- | --- | --- | --- | --- |
| D.Glucosamine.6.Suflate | C02827 | 5.31E+04 ± 4.74E+02 | 77.86E+02 ± 1.80E+02 | 7969.16 | <0.01 | High CO_2_ |
| 4.Hydroxybenzaldehyde | C00633 | 41.61E+02 ± 1.88E+02 | 11.03E+02 ± 3.06E+02 | 72.33 | <0.01 | High CO_2_ |
| Acetoacetate | C00164 | 6.94E+04 ± 17.89E+02 | 5.17E+04 ± 15.66E+02 | 55.09 | <0.01 | High CO_2_ |
| S.1.Phenylethanol | C07112 | 74.09E+04 ± 3.37E+04 | 48.96E+04 ± 3.52E+04 | 26.59 | 0.01 | High CO_2_ |
| Shikimate | C00493 | 66.48E+04 ± 7.94E+04 | 28.77E+04 ± 2.28E+04 | 20.85 | 0.01 | High CO_2_ |
| Gallic.Acid | C01424 | 69.77E+02 ± 8.10E+02 | 23.49E+02 ± 7.62E+02 | 17.31 | 0.01 | High CO_2_ |
| L.Proline | C16435 | 79.17E+06 ± 2.13E+06 | 46.84E+06 ± 8.59E+06 | 13.33 | 0.02 | High CO_2_ |
| D.Arabinose | C00216 | 78.30E+04 ± 62.38E+02 | 69.28E+04 ± 3.15E+04 | 7.88 | 0.05 | High CO_2_ |
| Fisetin | C10041 | 1.19E+08 ± 13.65E+06 | 80.10E+06 ± 5.36E+06 | 6.95 | 0.06 | High CO_2_ |
| Salicylate | C00805 | 55.13E+02 ± 8.60E+02 | 25.02E+02 ± 7.96E+02 | 6.60 | 0.06 | High CO_2_ |
| Adenosine.5.Monophosphate | C00020 | 12.59E+04 ± 2.09E+04 | 5.87E+04 ± 2.20E+04 | 4.88 | 0.09 | High CO_2_ |
| Myo.Inositol | C00137 | 47.75E+06 ± 5.72E+06 | 32.52E+06 ± 3.90E+06 | 4.84 | 0.09 | High CO_2_ |
| Arabitol | C01904 | 56.96E+04 ± 5.56E+04 | 42.47E+04 ± 3.60E+04 | 4.79 | 0.09 | High CO_2_ |
| 4.Aminobutanoate (GABA) | C00334 | 35.74E+04 ± 1.62E+04 | 32.04E+04 ± 49.08E+02 | 4.78 | 0.09 | High CO_2_ |
| L.Sorbose | C00247 | 38.14E+06 ± 13.90E+06 | 8.06E+06 ± 88.34E+04 | 4.67 | 0.10 | High CO_2_ |
| Sucrose | C00089 | 1.26E+08 ± 6.37E+06 | 1.03E+08 ± 9.44E+06 | 4.19 | 0.11 | High CO_2_ |
| Fructose | C00095 | 56.19E+06 ± 22.86E+06 | 13.02E+06 ± 1.54E+06 | 3.55 | 0.13 | High CO_2_ |
| D.Mannose | C00159 | 1.85E+06 ± 72.02E+04 | 53.57E+04 ± 3.74E+04 | 3.33 | 0.14 | High CO_2_ |
| Diethanolamine | C06772 | 6.78E+04 ± 3.31E+04 | 75.22E+02 ± 39.43E+02 | 3.26 | 0.15 | High CO_2_ |
| Phloroglucinol | C02183 | 7.62E+06 ± 1.29E+06 | 5.25E+06 ± 45.37E+04 | 3.02 | 0.16 | High CO_2_ |
| Succinate.Semialdehyde | C00232 | 1.16E+04 ± 30.63E+02 | 62.22E+02 ± 5.92E+02 | 2.99 | 0.16 | High CO_2_ |
| D.Lyxosylamine |  | 1.91E+06 ± 31.60E+04 | 1.33E+06 ± 12.64E+04 | 2.85 | 0.17 | High CO_2_ |
| 5.Methylthioadenosine | C00170 | 4.48E+04 ± 22.82E+02 | 2.62E+04 ± 1.08E+04 | 2.85 | 0.17 | High CO_2_ |
| D.Trehalose | C01083 | 1.79E+06 ± 25.93E+04 | 1.13E+06 ± 31.01E+04 | 2.63 | 0.18 | High CO_2_ |
| Rosmarinic.Acid | C01850 | 57.90E+04 ± 33.30E+04 | 4.21E+04 ± 2.40E+04 | 2.59 | 0.18 | High CO_2_ |
| Monoshaccharides Hexoses |  | 1.86E+06 ± 77.52E+04 | 61.80E+04 ± 4.49E+04 | 2.55 | 0.19 | High CO_2_ |
| Creatine | C00300 | 81.76E+04 ± 47.44E+04 | 7.15E+04 ± 3.75E+04 | 2.46 | 0.19 | High CO_2_ |
| L.Tyrosine | C01536 | 35.30E+04 ± 4.75E+04 | 28.04E+04 ± 43.56E+02 | 2.32 | 0.20 | High CO_2_ |
| Pyridoxine | C00314 | 16.31E+04 ± 1.99E+04 | 11.70E+04 ± 2.41E+04 | 2.17 | 0.21 | High CO_2_ |
| Monosaccharides Pentoses |  | 12.64E+04 ± 1.84E+04 | 9.74E+04 ± 90.09E+02 | 2.00 | 0.23 | High CO_2_ |
| 4.Guanidinobutanoate | C01035 | 22.37E+04 ± 11.96E+04 | 5.41E+04 ± 2.22E+04 | 1.94 | 0.24 | High CO_2_ |
| L.Pipecolic.Acid | C00408 | 1.49E+06 ± 37.59E+04 | 95.68E+04 ± 6.52E+04 | 1.94 | 0.24 | High CO_2_ |
| Hypoxanthine | C00262 | 15.34E+04 ± 6.12E+04 | 6.68E+04 ± 1.33E+04 | 1.91 | 0.24 | High CO_2_ |
| Creatinine | C00791 | 11.50E+04 ± 5.92E+04 | 3.48E+04 ± 67.41E+02 | 1.81 | 0.25 | High CO_2_ |
| N.Acetyl.Dl.Glutamic.Acid | C00624 | 2.06E+06 ± 34.20E+04 | 1.40E+06 ± 37.90E+04 | 1.69 | 0.26 | High CO_2_ |
| L.Valine | C00183 | 13.72E+06 ± 7.03E+06 | 4.33E+06 ± 2.19E+06 | 1.63 | 0.27 | High CO_2_ |
| Adenosine | C00212 | 23.58E+04 ± 13.25E+04 | 7.69E+04 ± 1.04E+04 | 1.43 | 0.30 | High CO_2_ |
| Pyruvic.Aldehyde | C00546 | 20.55E+04 ± 9.51E+04 | 10.21E+04 ± 1.06E+04 | 1.17 | 0.34 | High CO_2_ |
| Caffeic.Acid | C01197 | 87.49E+04 ± 20.75E+04 | 65.42E+04 ± 4.94E+04 | 1.07 | 0.36 | High CO_2_ |
| 6.Phosphogluconic.Acid | C00345 | 7.33E+04 ± 2.22E+04 | 5.00E+04 ± 58.99E+02 | 1.03 | 0.37 | High CO_2_ |
| Sugars.Alcohol.Hexoses |  | 96.95E+02 ± 13.34E+02 | 75.83E+02 ± 17.26E+02 | 0.94 | 0.39 | High CO_2_ |
| L.Isoleucine | C16434 | 1.97E+06 ± 55.89E+04 | 1.41E+06 ± 18.19E+04 | 0.93 | 0.39 | High CO_2_ |
| D.Pantothenic.Acid | C00864 | 10.41E+04 ± 3.41E+04 | 6.96E+04 ± 1.51E+04 | 0.86 | 0.41 | High CO_2_ |
| Xylitol | C00379 | 10.81E+04 ± 2.55E+04 | 7.99E+04 ± 1.90E+04 | 0.78 | 0.43 | High CO_2_ |
| 4.Hydroxy.L.Phenylglycine.Pyridoxal | CA1445 | 18.83E+04 ± 7.99E+04 | 12.21E+04 ± 47.18E+02 | 0.69 | 0.45 | High CO_2_ |
| 1.Aminocyclopropane.1.Carboxylate | C01234 | 2.29E+06 ± 12.23E+04 | 1.87E+06 ± 51.08E+04 | 0.63 | 0.47 | High CO_2_ |
| S.Malate | C00711 | 5.36E+06 ± 2.48E+06 | 3.31E+06 ± 1.07E+06 | 0.57 | 0.49 | High CO_2_ |
| Luteolin | C01514 | 6.77E+06 ± 3.80E+06 | 3.72E+06 ± 1.47E+06 | 0.56 | 0.50 | High CO_2_ |
| Galactitol | C01697 | 84.07E+02 ± 20.09E+02 | 60.72E+02 ± 24.15E+02 | 0.55 | 0.50 | High CO_2_ |
| 3.Methoxytyramine | C05587 | 5.19E+04 ± 9.72E+02 | 4.69E+04 ± 67.02E+02 | 0.53 | 0.51 | High CO_2_ |
| D.Gulonic.Acid.Gama.Lactone | C01040 | 3.69E+04 ± 1.04E+04 | 2.92E+04 ± 42.98E+02 | 0.47 | 0.53 | High CO_2_ |
| D.Malic.Acid | C00497 | 3.48E+06 ± 1.49E+06 | 2.43E+06 ± 85.22E+04 | 0.38 | 0.57 | High CO_2_ |
| Turanose | C19636 | 1.60E+06 ± 18.61E+04 | 1.41E+06 ± 30.46E+04 | 0.28 | 0.63 | High CO_2_ |
| Leucine | C16439 | 54.55E+04 ± 16.48E+04 | 44.94E+04 ± 8.92E+04 | 0.26 | 0.63 | High CO_2_ |
| L.DOPA | C00355 | 52.57E+04 ± 23.02E+04 | 39.15E+04 ± 10.68E+04 | 0.19 | 0.69 | High CO_2_ |
| Phenylacetic.Acid | C07086 | 41.63E+02 ± 23.39E+02 | 27.12E+02 ± 26.01E+02 | 0.17 | 0.70 | High CO_2_ |
| 3.Hydroxykynurenine | C02794 | 8.44E+04 ± 3.04E+04 | 7.02E+04 ± 1.69E+04 | 0.17 | 0.70 | High CO_2_ |
| Glutaric.Acid | C00489 | 11.32E+04 ± 2.64E+04 | 10.32E+04 ± 52.65E+02 | 0.14 | 0.73 | High CO_2_ |
| Resorcinol.Monoacetate | C12064 | 88.44E+02 ± 29.12E+02 | 74.55E+02 ± 23.71E+02 | 0.14 | 0.73 | High CO_2_ |
| Rs.Mevalonic.Acid | C00418 | 51.14E+02 ± 4.67E+02 | 46.72E+02 ± 12.80E+02 | 0.11 | 0.76 | High CO_2_ |
| Mandelic.Acid | C01984 | 2.10E+04 ± 85.77E+02 | 1.82E+04 ± 41.11E+02 | 0.09 | 0.78 | High CO_2_ |
| 5.Methylcytosine.Hydrocloride | C02376 | 5.16E+04 ± 89.69E+02 | 4.92E+04 ± 43.70E+02 | 0.05 | 0.83 | High CO_2_ |
| Nicotinamide | C00153 | 1.11E+06 ± 6.34E+04 | 1.09E+06 ± 15.29E+04 | 0.02 | 0.89 | High CO_2_ |
| L.Phenylalanine | C02057 | 75.89E+04 ± 14.95E+04 | 73.54E+04 ± 7.22E+04 | 0.02 | 0.89 | High CO_2_ |
| Naringenin | C00509 | 22.80E+02 ± 9.49E+02 | 22.46E+02 ± 16.78E+02 | 0.00 | 0.99 | High CO_2_ |
|  |  |  |  |  |  |  |
| a.Ketoglutaric.Acid | C00026 | 1.09E+04 ± 29.89E+02 | 6.06E+04 ± 27.71E+02 | 148.52 | <0.01 | Low CO_2_ |
| D.3.Phosphoglyceric.Acid (Glycerate 3P) | C00597 | 95.48E+02 ± 53.36E+02 | 7.63E+04 ± 78.57E+02 | 49.41 | <0.01 | Low CO_2_ |
| O.Succinyl.L.Homoserine | C01118 | 22.50E+04 ± 2.74E+04 | 1.03E+06 ± 17.17E+04 | 21.50 | 0.01 | Low CO_2_ |
| 1.Methyladenine | C02216 | 3.62E+04 ± 93.22E+02 | 7.93E+04 ± 52.28E+02 | 16.24 | 0.02 | Low CO_2_ |
| Glutamic.Acid (Glutamate) | C00025 | 3.90E+06 ± 45.34E+04 | 8.71E+06 ± 1.20E+06 | 14.10 | 0.02 | Low CO_2_ |
| Succinate | C00042 | 6.40E+04 ± 38.40E+02 | 13.28E+04 ± 1.81E+04 | 13.82 | 0.02 | Low CO_2_ |
| Nalpha.Acetyl.L.Lysine | C12989 | 3.50E+04 ± 84.95E+02 | 7.55E+04 ± 95.55E+02 | 10.04 | 0.03 | Low CO_2_ |
| Maleamate | C01596 | 3.00E+04 ± 51.71E+02 | 8.13E+04 ± 2.14E+04 | 5.42 | 0.08 | Low CO_2_ |
| 2.Hydroxypyridine | C02502 | 77.64E+04 ± 7.79E+04 | 1.27E+06 ± 19.79E+04 | 5.36 | 0.08 | Low CO_2_ |
| Pyruvate | C00022 | 5.69E+04 ± 20.32E+02 | 7.38E+04 ± 69.77E+02 | 5.35 | 0.08 | Low CO_2_ |
| Guanosine | C00387 | 3.00E+04 ± 24.28E+02 | 5.57E+04 ± 1.11E+04 | 5.18 | 0.09 | Low CO_2_ |
| Sugars.Alcohol.Pentoses |  | 37.90E+02 ± 9.48E+02 | 83.83E+02 ± 18.06E+02 | 5.07 | 0.09 | Low CO_2_ |
| Quinoline | C06413 | 3.37E+04 ± 75.35E+02 | 4.99E+04 ± 16.23E+02 | 4.42 | 0.10 | Low CO_2_ |
| 3.Dehydroshikimate | C02637 | 73.98E+02 ± 16.20E+02 | 1.16E+04 ± 11.51E+02 | 4.37 | 0.10 | Low CO_2_ |
| N.Acetyl.L.Alanine | C01073 | 2.98E+04 ± 3.52E+02 | 3.65E+04 ± 36.02E+02 | 3.45 | 0.14 | Low CO_2_ |
| Hexoses.Phosphate |  | 4.67E+04 ± 2.50E+04 | 11.35E+04 ± 2.73E+04 | 3.26 | 0.15 | Low CO_2_ |
| Nepsilon.Nepsilon.Nepsilon.Trimethyllysine | C03793 | 13.64E+02 ± 46.86E+00 | 34.27E+02 ± 14.85E+02 | 3.46 | 0.16 | Low CO_2_ |
| Urocanate | C00785 | 3.73E+04 ± 34.12E+02 | 14.66E+04 ± 6.40E+04 | 2.91 | 0.16 | Low CO_2_ |
| Uracil | C00106 | 16.81E+04 ± 91.72E+02 | 25.08E+04 ± 4.78E+04 | 2.88 | 0.16 | Low CO_2_ |
| Pyridoxamine | C00534 | 3.63E+04 ± 9.68E+02 | 5.83E+04 ± 1.37E+04 | 2.55 | 0.19 | Low CO_2_ |
| D.Glucuronolactone | C00191 | 8.65E+04 ± 2.41E+04 | 12.34E+04 ± 42.83E+02 | 2.27 | 0.21 | Low CO_2_ |
| Azelaic.Acid | C08261 | 34.55E+02 ± 8.16E+02 | 64.63E+02 ± 18.57E+02 | 2.20 | 0.21 | Low CO_2_ |
| Histamine | C00388 | 4.76E+04 ± 59.27E+02 | 6.39E+04 ± 95.39E+02 | 2.12 | 0.22 | Low CO_2_ |
| Guanine | C00242 | 54.94E+04 ± 2.76E+04 | 92.49E+04 ± 26.23E+04 | 2.03 | 0.23 | Low CO_2_ |
| N.Acetylglycine | CA1212 | 6.57E+04 ± 86.22E+02 | 9.12E+04 ± 1.71E+04 | 1.78 | 0.25 | Low CO_2_ |
| Adenine | C00147 | 7.66E+06 ± 56.81E+04 | 9.48E+06 ± 1.24E+06 | 1.76 | 0.25 | Low CO_2_ |
| 4.Hydroxy.L.Proline | C01157 | 10.72E+04 ± 71.02E+02 | 12.54E+04 ± 1.18E+04 | 1.73 | 0.26 | Low CO_2_ |
| Citrate | C00158 | 2.11E+06 ± 87.25E+04 | 3.54E+06 ± 66.92E+04 | 1.69 | 0.26 | Low CO_2_ |
| Cytosine | C00380 | 41.57E+04 ± 3.66E+04 | 60.80E+04 ± 14.70E+04 | 1.61 | 0.27 | Low CO_2_ |
| Salsolinol | C09642 | 3.05E+04 ± 17.30E+02 | 4.47E+04 ± 1.12E+04 | 1.55 | 0.28 | Low CO_2_ |
| N.Acetyl.D.Tryptophan | C03137 | 63.51E+02 ± 68.60E+00 | 1.12E+04 ± 39.55E+02 | 1.52 | 0.29 | Low CO_2_ |
| 3.Aminoisobutanoate | C05145 | 1.72E+04 ± 44.18E+02 | 2.32E+04 ± 27.27E+02 | 1.31 | 0.32 | Low CO_2_ |
| Formononetin | C00858 | 3.47E+02 ± 89.93E+00 | 6.55E+02 ± 2.59E+02 | 1.26 | 0.33 | Low CO_2_ |
| L.Alanine | C00041 | 60.19E+04 ± 4.15E+04 | 76.48E+04 ± 14.37E+04 | 1.19 | 0.34 | Low CO_2_ |
| Uridine | C00299 | 4.03E+04 ± 1.17E+04 | 5.69E+04 ± 99.28E+02 | 1.17 | 0.34 | Low CO_2_ |
| Alpha.Aminoadipate | C00956 | 10.57E+04 ± 89.31E+02 | 13.83E+04 ± 2.92E+04 | 1.14 | 0.35 | Low CO_2_ |
| 2.6.Dihydroxypyridine | C03056 | 5.05E+04 ± 50.38E+02 | 6.49E+04 ± 1.26E+04 | 1.13 | 0.35 | Low CO_2_ |
| 3.2.Hydroxyphenyl.Propanoate | C01198 | 59.96E+02 ± 5.67E+02 | 80.47E+02 ± 18.64E+02 | 1.11 | 0.35 | Low CO_2_ |
| L.Serine | C00716 | 16.83E+04 ± 70.83E+02 | 24.18E+04 ± 7.67E+04 | 0.91 | 0.39 | Low CO_2_ |
| L.Threonine | C00188 | 18.64E+04 ± 3.22E+04 | 24.26E+04 ± 5.02E+04 | 0.89 | 0.40 | Low CO_2_ |
| Palmitic.Acid | C00249 | 2.45E+06 ± 4.92E+04 | 3.25E+06 ± 99.43E+04 | 0.65 | 0.47 | Low CO_2_ |
| Disaccharides |  | 3.44E+06 ± 49.72E+04 | 4.03E+06 ± 55.30E+04 | 0.63 | 0.47 | Low CO_2_ |
| Eriodictyol | C05631 | 11.96E+02 ± 5.42E+02 | 26.31E+02 ± 20.88E+02 | 0.44 | 0.54 | Low CO_2_ |
| Tyramine | C00483 | 4.08E+04 ± 95.01E+02 | 5.21E+04 ± 1.42E+04 | 0.44 | 0.54 | Low CO_2_ |
| Dehydroascorbate | C05422 | 56.00E+04 ± 26.29E+04 | 72.58E+04 ± 2.23E+04 | 0.40 | 0.56 | Low CO_2_ |
| Linoleic.Acid | C01595 | 18.06E+04 ± 5.69E+04 | 29.20E+04 ± 21.40E+04 | 0.40 | 0.57 | Low CO_2_ |
| 5.Oxo.L.Proline | C01879 | 8.52E+06 ± 96.91E+04 | 11.23E+06 ± 4.36E+06 | 0.37 | 0.58 | Low CO_2_ |
| Deoxy-Hexoses |  | 60.58E+02 ± 13.94E+02 | 70.73E+02 ± 10.30E+02 | 0.34 | 0.59 | Low CO_2_ |
| 1.2.Phenylenediamine | C14402 | 4.87E+04 ± 1.21E+04 | 5.86E+04 ± 1.49E+04 | 0.27 | 0.63 | Low CO_2_ |
| 6.Hydroxynicotinate | C01020 | 4.87E+04 ± 48.38E+02 | 5.34E+04 ± 75.80E+02 | 0.27 | 0.63 | Low CO_2_ |
| 3.Amino.5.Hydroxybenzoic.Acid | C12107 | 3.48E+04 ± 18.80E+02 | 3.78E+04 ± 60.36E+02 | 0.23 | 0.66 | Low CO_2_ |
| L.Glutamine | C00303 | 14.20E+06 ± 2.30E+06 | 18.46E+06 ± 9.55E+06 | 0.19 | 0.69 | Low CO_2_ |
| Glyceraldehyde | C02154 | 40.84E+04 ± 5.64E+04 | 44.02E+04 ± 4.72E+04 | 0.19 | 0.69 | Low CO_2_ |
| Fumarate | C00122 | 15.47E+04 ± 5.17E+04 | 18.25E+04 ± 3.90E+04 | 0.18 | 0.69 | Low CO_2_ |
| Trigonelline | C01004 | 6.00E+06 ± 1.24E+06 | 7.09E+06 ± 2.27E+06 | 0.18 | 0.70 | Low CO_2_ |
| L.Asparagine | C16438 | 11.44E+04 ± 1.79E+04 | 14.46E+04 ± 7.41E+04 | 0.16 | 0.71 | Low CO_2_ |
| Glycerol.3.Phosphate | C00093 | 68.27E+04 ± 15.36E+04 | 76.28E+04 ± 13.98E+04 | 0.15 | 0.72 | Low CO_2_ |
| Galactonic.Acid | C00880 | 48.72E+04 ± 15.57E+04 | 59.85E+04 ± 26.28E+04 | 0.13 | 0.73 | Low CO_2_ |
| Aspartate | C00049 | 1.27E+06 ± 47.44E+04 | 1.43E+06 ± 9.70E+04 | 0.11 | 0.76 | Low CO_2_ |
| Thymine | C00178 | 7.87E+04 ± 76.41E+02 | 8.34E+04 ± 1.38E+04 | 0.09 | 0.78 | Low CO_2_ |
| Glyceric.Acid | C00258 | 18.83E+04 ± 3.55E+04 | 19.75E+04 ± 1.13E+04 | 0.06 | 0.82 | Low CO_2_ |
| 3.Amino.4.Hydroxybenzoic.Acid | C12115 | 4.23E+04 ± 96.25E+02 | 4.66E+04 ± 2.06E+04 | 0.04 | 0.86 | Low CO_2_ |
| Amino-Sugars |  | 5.63E+04 ± 3.52E+04 | 6.37E+04 ± 2.19E+04 | 0.03 | 0.87 | Low CO_2_ |
| Nicotinate.Picolinic.Acid | C00253 | 4.84E+04 ± 61.66E+02 | 4.96E+04 ± 52.35E+02 | 0.02 | 0.89 | Low CO_2_ |
| L.Arginine | C00062 | 1.00E+04 ± 24.94E+02 | 1.07E+04 ± 43.58E+02 | 0.01 | 0.91 | Low CO_2_ |
| 2.Aminophenol | C01987 | 72.94E+04 ± 5.98E+04 | 74.03E+04 ± 12.21E+04 | 0.01 | 0.94 | Low CO_2_ |
| 4.Acetamidobutanoate | C02946 | 11.80E+04 ± 2.17E+04 | 11.89E+04 ± 80.55E+02 | 0.00 | 0.97 | Low CO_2_ |

## LC-MS and GC-MS Parameters

**Table S11**. Parameters applied to GC-MS chromatograms with Metabolite Detector 2.5 for the obtaining of the metabolomic profiles of Eelgrass.

| **Tool settings** | | |
| --- | --- | --- |
| Centroid | Threshold begin | 10 |
|  | Peak threshold end | -5 |
|  | Maximal baseline | 30 |
|  | FWHM | 0.1 |
| Deconvolution | Peak threshold | 10 |
|  | Minimum peak height | 10 |
|  | Deconvolution width (scans) | 8 |
| Identification | Max RI difference | 20 |
|  | Cutoff score | 0.6 |
|  | Pure/Impure | 0.6 |
|  | Scaled lib | Yes |
|  | Combined score | Yes |
| Quantification | Minimal distance | 0.5 |
|  | Minimal required quality index | 1 |
|  | Exclude | 72.5 to 73.5  146.5 to 147.5 |
|  | | |
| **Batch quantification Settings** | | |
| Compound matching | ARI | 20 |
|  | Pure/Impure | 0.6 |
|  | Req. Score | 0.6 |
|  | RI+Spec | OK |
| Identification | ARI | 20 |
|  | Pure/Impure | 0.6 |
|  | RI+Spec | OK |
| Other settings | Compound reproducibility | 0 |
|  | Max. Peak drisc. index | 100 |
|  | S/N | 15 |
|  | Number of ions | 4 |
|  | Extended SIC Scan | Yes |

**Table S12.** Score, retention index (RI), retention time (RT) and signal to noise ratio (S/N) of the matched metabolites in GC-MS chromatograms processed with Metabolite Detector 2.5.

|  | Score | RT Standard  (min) | Measured Avg. RT (Min) | Avg. S/N | Considered for the study |
| --- | --- | --- | --- | --- | --- |
| a.ketoglutaric.acid | 0.86 | 13.85 | 13.91 | 14.43 | YES |
| Arabitol | 0.85 | 15.60 | 15.5 | 47.47 | YES |
| Caffeic.acid | 0.98 | 19.75 | 19.82 | 66.07 | YES |
| Citric.acid | 0.9 | 16.83 | 16.77 | 84.86 | YES |
| D.Arabinose | 0.89 | 15.19 | 15.28 | 22.82 | YES |
| D.Lyxosylamine | 0.91 | 14.73 | 14.74 | 89.01 | YES |
| D.Malic.acid | 0.92 | 12.79 | 12.85 | 143.04 | YES |
| D.Mannose | 0.96 | 17.66 | 17.72 | 77.96 | YES |
| D.Trehalose | 0.81 | 25.20 | 25.22 | 74.59 | YES |
| Fructose | 0.89 | 17.28 | 17.44 | 458.08 | YES |
| Galactonic.acid | 0.88 | 18.77 | 18.73 | 45 | YES |
| Glyceric.acid | 0.94 | 10.73 | 10.78 | 26.84 | YES |
| Glycerol.3.phosphate | 0.93 | 16.05 | 16.17 | 58.68 | YES |
| Glycine | 0.99 | 10.45 | 10.44 | 26.99 | YES |
| L.DOPA | 0.76 | 19.08 | 19.24 | 107.04 | YES |
| L.Glutamic.acid | 0.86 | 13.33 | 13.34 | 22.99 | YES |
| L.Glutamic.acid | 0.93 | 13.23 | 13.27 | 147.83 | YES |
| L.proline | 0.96 | 10.32 | 10.3 | 128.18 | YES |
| L.sorbose | 0.72 | 17.23 | 17.55 | 364.34 | YES |
| Linoleic.acid | 0.84 | 20.39 | 20.4 | 11.92 | YES |
| Myo.inositol | 0.93 | 19.70 | 19.62 | 604.44 | YES |
| N.acetyl.L.glutamic.acid | 0.62 | 13.06 | 12.94 | 66.37 | YES |
| Palmitic.acid | 0.93 | 18.84 | 18.86 | 104.36 | YES |
| Shikimic.acid | 0.84 | 16.43 | 16.6 | 23.86 | YES |
| Sucrose | 0.93 | 24.41 | 24.36 | 707.99 | YES |
| Turanose | 0.77 | 24.81 | 24.76 | 67.47 | YES |
| 2.Hydroxybutyric.acid | 0.89 | 7.85 | 7.92 | 19.86 | NO |
| 4.Hydroxybenzoic.acid | 0.81 | 14.50 | 14.49 | 32.52 | NO |
| Arbutin | 0.79 | 23.39 | 23.37 | 20.87 | NO |
| Coniferyl.alcohol | 0.62 | 17.97 | 18 | 10.59 | NO |
| D.Gluconic.acid | 0.83 | 18.31 | 18.4 | 10.58 | NO |
| D.Glucose | 0.77 | 17.98 | 18.07 | 51.68 | NO |
| D.Glucuronic.acid | 0.65 | 18.15 | 18.13 | 16.73 | NO |
| D.Sorbitol | 0.8 | 17.89 | 17.91 | 23.75 | NO |
| Dehydroascorbic.acid | 0.81 | 18.01 | 18.28 | 4 | NO |
| L.glutamine | 0.71 | 12.71 | 12.66 | 21.23 | NO |
| Lactulose | 0.78 | 23.86 | 23.88 | 82.46 | NO |
| Lactulose | 0.67 | 24.43 | 24.23 | 33.02 | NO |
| Methyl.beta.D.galactopyranoside | 0.64 | 16.93 | 16.87 | 31.65 | NO |
| N.acetyl.L.cysteine | 0.75 | 15.24 | 15.27 | 14.99 | NO |
| Norvaline | 0.92 | 9.46 | 9.15 | 12.02 | NO |
| Ribitol | 0.84 | 15.66 | 15.67 | 6.21 | NO |
| Ribonic.acid.gamma.lactone | 0.61 | 15.05 | 14.64 | 22.47 | NO |
| Rosmarinic.acid | 0.63 | 29.69 | 29.51 | 12.71 | NO |
| Scyllo.inositol | 0.82 | 19.10 | 18.99 | 9.02 | NO |
| Sialic.acid | 0.61 | 22.25 | 22.54 | 42.45 | NO |

**Table S13.** Parameters applied to LC-MS RAW files with MZMine 2.26 (Pluskal et al., 2010) to obtain the metabolomic fingerprintings of eelgrass samples from both positive and negative ionization modes.

|  |  | (+H) Chromatograms |  | (-H) Chromatograms |
| --- | --- | --- | --- | --- |
| **1** | **Baseline correction – RollingBall baseline corrector** |  |  |  |
|  | Chromatogram type | TIC |  | TIC |
|  | Use m/z bins | No |  | No |
|  | wm | 25 |  | 25 |
|  | ws | 25 |  | 25 |
| **2** | **Mass detection** (exact Mass) |  |  |  |
|  | Noise level | 1 × 10^4^ |  | 1 × 10^3^ |
| **3** | **Chromatogram builder (ADAP)**^58^ |  |  |  |
|  | Min group size in num. of scans | 3 |  | 3 |
|  | Group intensity threshold | 1 × 10^4^ |  | 1 × 10^3^ |
|  | Min highest intensity | 1 × 10^5^ |  | 1 × 10^4^ |
|  | m/z tolerance | 0.0005 or 6ppm |  | 0.0005 or 6ppm |
| **4** | **Smoothing** |  |  |  |
|  | Filter width | 5 |  | 5 |
| **5** | **Chromatogram deconvolution (local minimum search)** |  |  |  |
|  | Chromatographic threshold | 40% |  | 40% |
|  | Search minimum in RT range (min) | 0.25 |  | 0.25 |
|  | Minimum relative height | 50% |  | 50% |
|  | Minimum absolute height | 1 × 10^4^ |  | 1 × 10^3^ |
|  | Minimum ratio of peak top/edge | 1.5 |  | 1.5 |
|  | Peak duration range | 0-2 min |  | 0-2 min |
| **6** | **Isotopic peak grouper** |  |  |  |
|  | m/z tolerance | 0.0005 or 6ppm |  | 0.0005 or 6ppm |
|  | Retention Time tolerance | 0.25 min |  | 0.25 min |
|  | Max charge | 1 |  | 1 |
|  | Representative isotope | Most intense |  | Most intense |
| **7** | **Retention Time Normalizer** |  |  |  |
|  | m/z tolerance | 0.0005 or 6ppm |  | 0.0005 or 6ppm |
|  | Retention Time tolerance | 0.25 min |  | 0.25 min |
|  | Minimum Standard Intensity | 1 × 10^5^ |  | 1 × 10^4^ |
| **8** | **Chromatogram alignment (join alignment)** |  |  |  |
|  | m/z tolerance | 0.0005 or 6ppm |  | 0.0005 or 6ppm |
|  | Weight for m/z | 80 |  | 80 |
|  | RT tolerance | 0.25 |  | 0.25 |
|  | Weight for RT | 20 |  | 20 |
| **7** | **Gap filling (Peak Finder)** |  |  |  |
|  | Intensity tolerance | 60% |  | 60% |
|  | m/z tolerance | 0.0005 or 6ppm |  | 0.0005 or 6ppm |
|  | Retention time tolerance | 0.2 |  | 0.2 |
|  | RT correction | Yes |  | Yes |
| **8** | **Metabolite Assignation** |  |  |  |
|  | m/z tolerance | 0.0005 or 6ppm |  | 0.0005 or 6ppm |
|  | RT tolerance | 0.25 |  | 0.25 |

RT, retention time; m/z, mass to charge ratio

**Table S14.** Retention time (RT) and mass to charge ratio (m/z) of the deconvoluted ions in both negative and positive ionization modes assigned to metabolites with MZmine v.2.26 for LC-MS chromatograms. The assignment of the metabolites was based on the exact mass and RT of standards. RT and m/z of the standards are shown in the table. Error of m/z and RT of assigned ions to metabolites respect the m/z and RT of standards are shown. After applying the chromatogram builder and deconvolution algorithms from MZmine, several ions with the same exact mass may have been separated into two or more independent deconvoluted peaks presenting slightly different retention times. The following table show all the peaks assigned to a molecular compound based on the exact mass of their parent ion (in negative or positive mode). In the main manuscript, all identified metabolic features assigned to a same metabolite were summed to finally have a single variable per metabolite.

|  | m/z and RT of each ion assigned | | | Measured m/z and RT from Standards. | | Error of m/z and RT (deconvoluted ions vs. Standard ions) | | |
| --- | --- | --- | --- | --- | --- | --- | --- | --- |
|  | in MZmine v.2.26 | | |  |  |  |  |  |
| Ionization mode | Name | m/z | RT | m/z | RT | m/z | m/z | RT |
|  |  |  |  |  |  | (absolute) | (ppm) |  |
| POS | 1.2.Phenylenediamine | 109.08 | 1.41 | 109.08 | 1.35 | 0.00 | 0.42 | -0.06 |
| POS | 1.AMINOCYCLOPROPANE.1.CARBOXYLATE | 102.05 | 1.42 | 102.06 | 1.29 | 0.00 | 1.33 | -0.13 |
| POS | 1.METHYL.6.7.DIHYDROXY.1.2.3.4.TETRAHYDROISOQUINOLINE | 180.10 | 1.42 | 180.10 | 1.39 | 0.00 | 0.84 | -0.04 |
| POS | 1.Methyladenine.3.METHYLADENINE | 150.08 | 1.40 | 150.08 | 1.30 | 0.00 | 1.24 | -0.11 |
| POS | 1.PHENYLETHANOL | 123.08 | 11.95 | 123.08 | 11.90 | 0.00 | 1.59 | -0.05 |
| POS | 1.PHENYLETHANOL | 123.08 | 12.01 | 123.08 | 11.90 | 0.00 | 1.67 | -0.11 |
| POS | 2.6.DIHYDROXYPYRIDINE | 112.04 | 1.41 | 112.04 | 1.41 | 0.00 | 0.77 | 0.00 |
| POS | 2.AMINOPHENOL | 110.06 | 1.41 | 110.06 | 1.39 | 0.00 | 0.78 | -0.03 |
| POS | 2.HYDROXYPYRIDINE | 96.04 | 1.39 | 96.04 | 1.39 | 0.00 | 1.73 | 0.00 |
| NEG | 3.2.HYDROXYPHENYL.PROPANOATE | 165.06 | 11.12 | 165.06 | 10.74 | 0.00 | 1.78 | -0.39 |
| POS | AMINO.HYDROXYBENZOIC.ACID | 154.05 | 1.43 | 154.05 | 1.39 | 0.00 | 0.69 | -0.05 |
| POS | AMINO.HYDROXYBENZOIC.ACID | 154.05 | 1.73 | 154.05 | 1.76 | 0.00 | 1.08 | 0.03 |
| NEG | 3.AMINOISOBUTANOATE | 102.06 | 1.35 | 102.06 | 1.27 | 0.00 | 0.63 | -0.08 |
|  | 2.AMINO.2.METHYLPROPANOATE |  |  |  |  |  |  |  |
| NEG | 3.DEHYDROSHIKIMATE | 171.03 | 1.76 | 171.03 | 1.45 | 0.00 | 0.61 | -0.31 |
| POS | 3.HYDROXYKYNURENINE | 225.09 | 1.43 | 225.09 | 1.40 | 0.00 | 0.07 | -0.04 |
| POS | 3.HYDROXYKYNURENINE | 225.09 | 1.74 | 225.09 | 1.85 | 0.00 | -0.28 | 0.11 |
| NEG | 3.METHOXY.4.HYDROXYMANDELATE | 197.05 | 2.31 | 197.05 | 2.30 | 0.00 | 1.85 | -0.01 |
| POS | 3.METHOXYTYRAMINE | 168.10 | 1.42 | 168.10 | 1.35 | 0.00 | 0.60 | -0.07 |
| NEG | 4.ACETAMIDOBUTANOATE | 144.07 | 1.43 | 144.07 | 1.38 | 0.00 | 1.28 | -0.06 |
| POS | 4.ACETAMIDOBUTANOATE | 146.08 | 1.41 | 146.08 | 1.38 | 0.00 | 0.38 | -0.04 |
| POS | AMINOBUTANOATE | 104.07 | 1.36 | 104.07 | 1.25 | 0.00 | 0.63 | -0.11 |
| POS | 4.GUANIDINOBUTANOATE | 146.09 | 1.40 | 146.09 | 1.32 | 0.00 | 0.18 | -0.09 |
| POS | 4.HYDROXY.L.PHENYLGLYCINE | 168.07 | 1.41 | 168.07 | 1.35 | 0.00 | 0.51 | -0.06 |
|  | PYRIDOXAL |  |  |  |  |  |  |  |
| POS | 4.HYDROXY.L.PROLINE | 132.07 | 1.42 | 132.07 | 1.26 | 0.00 | 1.26 | -0.16 |
| POS | 4.HYDROXYBENZALDEHYDE | 123.04 | 7.70 | 123.04 | 7.71 | 0.00 | 1.51 | 0.01 |
| POS | 5.METHYLCYTOSINE | 126.07 | 1.38 | 126.07 | 1.28 | 0.00 | 0.63 | -0.10 |
| POS | 5.METHYLTHIOADENOSINE | 298.10 | 2.74 | 298.10 | 2.86 | 0.00 | 0.39 | 0.12 |
| POS | 5.OXO.D.PROLINE | 130.05 | 1.36 | 130.05 | 1.38 | 0.00 | 0.82 | 0.02 |
| POS | 5.OXO.D.PROLINE | 130.05 | 1.72 | 130.05 | 1.78 | 0.00 | 1.20 | 0.06 |
| NEG | 5.OXO.L.PROLINE | 128.04 | 1.36 | 128.04 | 1.37 | 0.00 | 0.89 | 0.01 |
| NEG | 5.OXO.L.PROLINE | 128.04 | 1.75 | 128.04 | 1.37 | 0.00 | -0.20 | -0.38 |
| POS | 6.HYDROXYNICOTINATE | 140.03 | 1.74 | 140.03 | 1.84 | 0.00 | 1.19 | 0.10 |
| POS | 6.Phosphogluconic.acid | 277.03 | 1.42 | 277.03 | 1.55 | 0.00 | -0.63 | 0.13 |
| POS | ACETOACETATE | 103.04 | 1.75 | 103.04 | 1.82 | 0.00 | 1.81 | 0.07 |
| NEG | ADENINE | 134.05 | 1.34 | 134.05 | 1.33 | 0.00 | 1.00 | -0.01 |
| POS | ADENINE | 136.06 | 1.39 | 136.06 | 1.33 | 0.00 | 0.63 | -0.06 |
| POS | ADENOSINE | 268.10 | 1.41 | 268.10 | 1.38 | 0.00 | -0.16 | -0.03 |
| POS | ADENOSINE | 268.10 | 1.73 | 268.10 | 1.85 | 0.00 | -0.50 | 0.12 |
| POS | ADENOSINE.5.MONOPHOSPHATE | 348.07 | 1.42 | 348.07 | 1.49 | 0.00 | -2.88 | 0.07 |
| POS | ADENOSINE.5.MONOPHOSPHATE | 348.07 | 1.42 | 348.07 | 1.49 | 0.00 | -4.46 | 0.07 |
| NEG | ALPHA.AMINOADIPATE | 160.06 | 1.42 | 160.06 | 1.34 | 0.00 | 1.40 | -0.08 |
| POS | ALPHA.AMINOADIPATE | 162.08 | 1.42 | 162.08 | 1.34 | 0.00 | 0.47 | -0.08 |
| POS | Sugars-Hexoses-Phosphate | 261.04 | 1.43 | 261.04 | 1.54 | 0.00 | -0.36 | 0.11 |
| POS | AZELAIC.ACID | 189.11 | 11.07 | 189.11 | 11.30 | 0.00 | 0.82 | 0.23 |
| NEG | CITRATE | 191.02 | 1.73 | 191.02 | 1.48 | 0.00 | 1.38 | -0.25 |
| POS | CREATINE | 132.08 | 1.37 | 132.08 | 1.32 | 0.00 | 1.03 | -0.06 |
| POS | CREATININE | 114.07 | 1.40 | 114.07 | 1.27 | 0.00 | 0.14 | -0.14 |
| POS | CYTOSINE | 112.05 | 1.35 | 112.05 | 1.26 | 0.00 | 0.68 | -0.09 |
| POS | D.3.PHOSPHOGLYCERIC.ACID | 187.00 | 1.46 | 187.00 | 1.62 | 0.00 | 0.49 | 0.16 |
| POS | ASPARTATE | 134.04 | 1.38 | 134.04 | 1.41 | 0.00 | 0.79 | 0.03 |
| NEG | D.GLUCOSAMINE.6.SULFATE | 259.01 | 1.37 | 259.01 | 1.34 | 0.00 | 2.94 | -0.04 |
| NEG | D.GLUCURONOLACTONE | 193.04 | 1.37 | 193.04 | 1.36 | 0.00 | 2.02 | -0.01 |
| NEG | D.GLUCURONOLACTONE | 193.04 | 1.40 | 193.04 | 1.36 | 0.00 | 1.96 | -0.04 |
| NEG | D.GULONIC.ACID.GAMA.LACTONE | 177.04 | 1.39 | 177.04 | 1.36 | 0.00 | 1.89 | -0.04 |
| POS | Amino-Sugars-C8 | 180.09 | 1.41 | 180.09 | 1.21 | 0.00 | -5.10 | -0.21 |
| POS | D.PANTOTHENIC.ACID | 220.12 | 1.44 | 220.12 | 1.42 | 0.00 | 0.03 | -0.03 |
| NEG | D.SORBITOL.GALACTITOL | 181.07 | 1.33 | 181.07 | 1.31 | 0.00 | 2.07 | -0.02 |
| NEG | Sugars-Pentoses | 149.05 | 1.39 | 149.05 | 1.32 | 0.00 | 1.44 | -0.07 |
| NEG | DEHYDROASCORBATE | 173.01 | 1.41 | 173.01 | 1.39 | 0.00 | 2.16 | -0.02 |
| NEG | DEHYDROASCORBATE | 173.01 | 1.63 | 173.01 | 1.39 | 0.00 | 1.41 | -0.24 |
| POS | DEHYDROASCORBATE | 175.02 | 1.73 | 175.02 | 1.67 | 0.00 | 0.72 | -0.07 |
| POS | DIETHANOLAMINE | 106.09 | 1.34 | 106.09 | 1.24 | 0.00 | 2.22 | -0.10 |
| POS | Eriodictyol | 289.07 | 12.16 | 289.07 | 12.26 | 0.00 | 4.55 | 0.10 |
| POS | Fisetin | 287.05 | 10.10 | 287.06 | 10.13 | 0.00 | 0.93 | 0.03 |
| NEG | Formononetin | 267.07 | 14.35 | 267.07 | 14.28 | 0.00 | 3.12 | -0.07 |
| NEG | Formononetin | 267.07 | 14.59 | 267.07 | 14.28 | 0.00 | 2.49 | -0.31 |
| NEG | FUMARATE | 115.00 | 1.71 | 115.00 | 1.51 | 0.00 | -0.14 | -0.20 |
| POS | GALACTITOL | 183.09 | 1.43 | 183.09 | 1.35 | 0.00 | 2.98 | -0.08 |
| NEG | Gallic.acid | 169.01 | 1.47 | 169.01 | 1.40 | 0.00 | 2.33 | -0.07 |
| NEG | Glutamic.acid | 146.05 | 1.35 | 146.05 | 1.29 | 0.00 | 1.74 | -0.06 |
| POS | Glutamic.acid | 148.06 | 1.36 | 148.06 | 1.29 | 0.00 | 1.32 | -0.07 |
| NEG | GLUTARATE | 131.04 | 1.81 | 131.04 | 1.53 | 0.00 | -0.50 | -0.28 |
| NEG | GLUTARATE | 131.04 | 2.43 | 131.04 | 2.13 | 0.00 | -0.89 | -0.31 |
| NEG | GLUTARATE | 131.03 | 1.43 | 131.04 | 1.34 | 0.00 | 0.49 | -0.10 |
| NEG | GLYCERALDEHYDE | 89.02 | 1.72 | 89.02 | 1.39 | 0.00 | -0.74 | -0.33 |
| POS | GUANINE | 152.06 | 1.40 | 152.06 | 1.33 | 0.00 | 0.76 | -0.08 |
| POS | GUANOSINE | 284.10 | 1.43 | 284.10 | 1.36 | 0.00 | -0.15 | -0.08 |
| POS | HISTAMINE | 112.09 | 1.42 | 112.09 | 1.30 | 0.00 | 0.41 | -0.12 |
| POS | HYPOXANTHINE | 137.05 | 1.42 | 137.05 | 1.37 | 0.00 | 0.85 | -0.06 |
| NEG | L.ALANINE | 88.04 | 1.37 | 88.04 | 1.28 | 0.00 | -0.07 | -0.09 |
| POS | L.ALANINE | 90.05 | 1.35 | 90.05 | 1.28 | 0.00 | 2.40 | -0.07 |
| NEG | Sugars-Alcohol-Pentoses | 151.06 | 1.35 | 151.06 | 1.31 | 0.00 | 1.42 | -0.04 |
| POS | L.ARGININE | 175.12 | 1.34 | 175.12 | 1.25 | 0.00 | 0.83 | -0.09 |
| POS | L.ASPARAGINE | 133.06 | 1.37 | 133.06 | 1.31 | 0.00 | 0.95 | -0.06 |
| NEG | L.ASPARAGINE | 131.05 | 1.33 | 131.05 | 1.31 | 0.00 | 1.18 | -0.02 |
| NEG | L.GLUTAMINE | 145.06 | 1.32 | 145.06 | 1.32 | 0.00 | 1.48 | -0.01 |
| POS | L.GLUTAMINE | 147.08 | 1.36 | 147.08 | 1.32 | 0.00 | 0.65 | -0.05 |
| POS | L.ISOLEUCINE | 132.10 | 1.75 | 132.10 | 1.82 | 0.00 | 1.33 | 0.07 |
| POS | L.LEUCINE | 132.10 | 1.44 | 132.10 | 1.37 | 0.00 | 1.33 | -0.08 |
| POS | L.PHENYLALANINE | 166.09 | 1.43 | 166.09 | 1.36 | 0.00 | 0.58 | -0.08 |
| NEG | L.PHENYLALANINE | 164.07 | 2.19 | 164.07 | 2.19 | 0.00 | -0.04 | -0.01 |
| POS | L.PHENYLALANINE | 166.09 | 2.18 | 166.09 | 2.19 | 0.00 | 0.64 | 0.01 |
| POS | L.PIPECOLIC.ACID | 130.09 | 1.41 | 130.09 | 1.35 | 0.00 | 0.97 | -0.06 |
| NEG | L.PROLINE | 114.06 | 1.46 | 114.06 | 1.35 | 0.00 | 1.09 | -0.12 |
| POS | L.PROLINE | 116.07 | 1.37 | 116.07 | 1.35 | 0.00 | 1.17 | -0.03 |
| POS | L.PROLINE | 116.07 | 1.38 | 116.07 | 1.35 | 0.00 | -5.89 | -0.04 |
| NEG | Deoxy-Sugars-Hexoses | 163.06 | 1.40 | 163.06 | 1.33 | 0.00 | 1.59 | -0.07 |
| NEG | L.SERINE | 104.04 | 1.34 | 104.04 | 1.29 | 0.00 | 0.71 | -0.05 |
| POS | L.SERINE | 106.05 | 1.35 | 106.05 | 1.29 | 0.00 | 1.85 | -0.06 |
| POS | L.SERINE | 106.05 | 1.35 | 106.05 | 1.29 | 0.00 | 1.66 | -0.06 |
| POS | L.THREONINE | 120.07 | 1.35 | 120.07 | 1.31 | 0.00 | 1.38 | -0.04 |
| POS | L.TYROSINE | 182.08 | 1.42 | 182.08 | 1.37 | 0.00 | 0.36 | -0.06 |
| POS | L.TYROSINE | 182.08 | 1.73 | 182.08 | 1.84 | 0.00 | 0.64 | 0.11 |
| POS | L.VALINE | 118.09 | 1.40 | 118.09 | 1.28 | 0.00 | 2.00 | -0.12 |
| POS | Luteolin | 287.06 | 12.17 | 287.06 | 12.36 | 0.00 | -0.08 | 0.19 |
| POS | MALEAMATE | 116.03 | 1.38 | 116.03 | 1.44 | 0.00 | -0.81 | 0.06 |
| NEG | MANDELIC.ACID | 151.04 | 5.35 | 151.04 | 5.35 | 0.00 | -0.44 | 0.00 |
| NEG | Sugars-Disaccharides | 341.11 | 1.33 | 341.11 | 1.24 | 0.00 | 3.27 | -0.09 |
| NEG | Sugars-Hexoses | 179.06 | 1.34 | 179.06 | 1.29 | 0.00 | 2.03 | -0.05 |
| POS | N.ACETYL.D.TRYPTOPHAN | 247.11 | 10.27 | 247.11 | 10.29 | 0.00 | 0.31 | 0.02 |
| NEG | N.ACETYL.DL.GLUTAMIC.ACID | 188.06 | 1.40 | 188.06 | 1.39 | 0.00 | 1.67 | -0.02 |
| POS | N.ACETYL.DL.GLUTAMIC.ACID | 190.07 | 1.44 | 190.07 | 1.39 | 0.00 | 0.45 | -0.06 |
| POS | N.ACETYL.L.ALANINE | 132.07 | 1.73 | 132.07 | 1.82 | 0.00 | 0.88 | 0.09 |
| POS | N.ACETYLGLYCINE | 118.05 | 1.44 | 118.05 | 1.55 | 0.00 | 1.66 | 0.11 |
| POS | N.ACETYLGLYCINE | 118.05 | 1.74 | 118.05 | 1.55 | 0.00 | 1.49 | -0.19 |
| POS | NALPHA.ACETYL.L.LYSINE | 189.12 | 1.40 | 189.12 | 1.28 | 0.00 | 0.40 | -0.12 |
| POS | Naringenin | 273.08 | 13.23 | 273.08 | 13.31 | 0.00 | 0.39 | 0.08 |
| POS | NEPSILON.NEPSILON.NEPSILON.TRIMETHYLLYSINE | 189.16 | 1.31 | 189.16 | 1.30 | 0.00 | 0.51 | -0.01 |
| POS | NICOTINAMIDE | 123.06 | 1.41 | 123.06 | 1.36 | 0.00 | 0.78 | -0.05 |
| POS | NICOTINAMIDE | 123.06 | 1.73 | 123.06 | 1.71 | 0.00 | 1.02 | -0.02 |
| POS | NICOTINATE.PICOLINIC.ACID | 124.04 | 1.71 | 124.04 | 1.70 | 0.00 | 1.18 | -0.01 |
| NEG | O.SUCCINYL.L.HOMOSERINE | 218.07 | 1.37 | 218.07 | 1.36 | 0.00 | 3.55 | -0.02 |
| POS | O.SUCCINYL.L.HOMOSERINE | 220.08 | 1.42 | 220.08 | 1.36 | 0.00 | 0.35 | -0.07 |
| POS | O.SUCCINYL.L.HOMOSERINE | 220.08 | 1.42 | 220.08 | 1.36 | 0.00 | 0.25 | -0.07 |
| NEG | PHENYLACETIC.ACID | 135.05 | 4.00 | 135.05 | 3.82 | 0.00 | -0.86 | -0.18 |
| NEG | Phloroglucinol | 125.02 | 1.76 | 125.02 | 1.43 | 0.00 | -0.53 | -0.34 |
| POS | Phloroglucinol | 127.04 | 1.39 | 127.04 | 1.43 | 0.00 | 1.31 | 0.04 |
| POS | Phloroglucinol | 127.04 | 1.40 | 127.04 | 1.43 | 0.00 | 0.68 | 0.03 |
| NEG | Phloroglucinol | 125.02 | 2.33 | 125.02 | 1.88 | 0.00 | -0.61 | -0.46 |
| POS | Phloroglucinol | 127.04 | 1.72 | 127.04 | 1.88 | 0.00 | 1.07 | 0.16 |
| POS | PYRIDOXAMINE | 169.10 | 1.41 | 169.10 | 1.35 | 0.00 | 0.57 | -0.07 |
| POS | PYRIDOXINE | 170.08 | 1.41 | 170.08 | 1.29 | 0.00 | 0.62 | -0.13 |
| NEG | PYRUVATE | 87.01 | 1.70 | 87.01 | 1.67 | 0.00 | -1.91 | -0.03 |
| NEG | PYRUVIC.ALDEHYDE | 71.01 | 1.36 | 71.01 | 1.38 | 0.00 | -1.35 | 0.02 |
| NEG | PYRUVIC.ALDEHYDE | 71.01 | 1.41 | 71.01 | 1.38 | 0.00 | -1.63 | -0.03 |
| POS | QUINOLINE | 130.07 | 2.18 | 130.07 | 2.23 | 0.00 | 1.12 | 0.05 |
| NEG | RESORCINOL.MONOACETATE | 151.04 | 11.00 | 151.04 | 10.87 | 0.00 | 1.88 | -0.13 |
| POS | ROSMARINIC.ACID | 361.09 | 11.02 | 361.09 | 11.18 | 0.00 | -0.29 | 0.16 |
| NEG | Mevalonic.Acid | 147.07 | 1.91 | 147.07 | 1.78 | 0.00 | 0.10 | -0.13 |
| NEG | S.MALATE | 133.01 | 1.42 | 133.01 | 1.45 | 0.00 | 1.23 | 0.03 |
| POS | SALICYLATE | 139.04 | 11.40 | 139.04 | 11.39 | 0.00 | 1.55 | -0.01 |
| NEG | SHIKIMATE | 173.05 | 1.41 | 173.05 | 1.39 | 0.00 | 1.93 | -0.02 |
| NEG | SUCCINATE | 117.02 | 1.76 | 117.02 | 1.41 | 0.00 | 0.12 | -0.36 |
| NEG | SUCCINATE.SEMIALDEHYDE | 101.02 | 2.00 | 101.02 | 1.79 | 0.00 | -1.35 | -0.21 |
| POS | THYMINE | 127.05 | 1.74 | 127.05 | 1.85 | 0.00 | 1.23 | 0.11 |
| POS | TRIGONELLINE | 138.05 | 1.40 | 138.06 | 1.36 | 0.00 | 0.84 | -0.05 |
| POS | TYRAMINE | 138.09 | 1.41 | 138.09 | 1.34 | 0.00 | 1.42 | -0.07 |
| POS | URACIL | 113.03 | 1.42 | 113.03 | 1.37 | 0.00 | 0.58 | -0.05 |
| NEG | URIDINE | 243.06 | 1.78 | 243.06 | 1.37 | 0.00 | 1.95 | -0.42 |
| POS | URIDINE | 245.08 | 1.42 | 245.08 | 1.37 | 0.00 | -0.10 | -0.06 |
| POS | UROCANATE | 139.05 | 1.41 | 139.05 | 1.33 | 0.00 | 0.98 | -0.09 |

RT, retention time m/z, mass to charge ratio ppm, parts per million
